# Using single-cell chromatin accessibility sequencing to characterize CD4+ T cells from murine tissues

**DOI:** 10.3389/fimmu.2023.1232511

**Published:** 2023-10-16

**Authors:** Kathrin Luise Braband, Annekathrin Silvia Nedwed, Sara Salome Helbich, Malte Simon, Niklas Beumer, Benedikt Brors, Federico Marini, Michael Delacher

**Affiliations:** ^1^ Institute of Immunology, University Medical Center Mainz, Mainz, Germany; ^2^ Research Center for Immunotherapy (FZI), University Medical Center Mainz, Mainz, Germany; ^3^ Institute of Medical Biostatistics, Epidemiology and Informatics (IMBEI), University Medical Center Mainz, Mainz, Germany; ^4^ Faculty of Biosciences, Heidelberg University, Heidelberg, Germany; ^5^ Division of Applied Bioinformatics, German Cancer Research Center (DKFZ), Heidelberg, Germany; ^6^ DKFZ-Hector Cancer Institute, University Medical Center Mannheim, Mannheim, Germany; ^7^ Division of Personalized Medical Oncology (A420), German Cancer Research Center (DKFZ), Heidelberg, Germany; ^8^ Department of Personalized Oncology, University Hospital Mannheim, Medical Faculty Mannheim, University of Heidelberg, Mannheim, Germany; ^9^ National Center for Tumor Diseases (NCT), Heidelberg, Germany; ^10^ German Cancer Consortium (DKTK), German Cancer Research Center (DKFZ), Heidelberg, Germany

**Keywords:** scATAC-seq, T cell isolation, tissue digestion, ArchR, Signac

## Abstract

The Assay for Transposase-Accessible Chromatin using sequencing (ATAC-seq) is a cutting-edge technology that enables researchers to assess genome-wide chromatin accessibility and to characterize cell type specific gene-regulatory programs. Recent technological progress allows for using this technology also on the single-cell level. In this article, we describe the whole value chain from the isolation of T cells from murine tissues to a complete bioinformatic analysis workflow. We start with methods for isolating scATAC-seq-ready CD4+ T cells from murine tissues such as visceral adipose tissue, skin, colon, and secondary lymphoid tissues such as the spleen. We describe the preparation of nuclei and quality control parameters during library preparation. Based on publicly available sequencing data that was generated using these protocols, we describe a step-by-step bioinformatic analysis pipeline for data pre-processing and downstream analysis. Our analysis workflow will follow the R-based bioinformatics framework ArchR, which is currently well established for scATAC-seq datasets. All in all, this work serves as a one-stop shop for generating and analyzing chromatin accessibility landscapes in T cells.

## Introduction

Chromatin describes DNA which is organized around histones and which makes up the structure of chromosomes. The accessibility of certain regions of chromatin is dependent on DNA methylation and histone modifications such as acetylation, phosphorylation or methylation (1, 2). The accessibility of chromatin to regulatory proteins such as transcription factors (TF) plays a key role in gene regulation. Analyzing chromatin accessibility in different cell types or disease states can help us gain a better understanding of the molecular programs that are active in the respective cell type or disease state, and can help elucidate the molecular mechanisms underlying the development of a certain disease.

Assay for Transposase-Accessible Chromatin using sequencing (ATAC-seq) was first introduced by Buenrostro et al. as a method for characterizing chromatin accessibility across the genome (3). ATAC-seq utilizes the hyperactive Tn5 transposase, which inserts sequencing adapters into regions of accessible chromatin. Sequencing of these accessible, or biologically active, regions lets us infer the cells’ identity (3, 4). For the characterization of cells in a heterogeneous population, single-cell (sc)ATAC-seq was developed. To this end, single-cells are separated and barcoded, treated with Tn5 transposase (prior to or after separation, depending on the technology), followed by library preparation. Different methods have been developed for achieving single-cell resolution, including combinatorial cellular indexing (5), nano-well technologies (6) and microfluidics platforms (7). Given the unique perspective provided into the regulatory mechanisms at the single-cell level, (sc)ATAC-seq is a valuable tool for characterizing cells from tissues. scATAC-seq and scRNA-seq are often used as complementary technologies, delivering a comprehensive picture of the cell identity that integrates transcriptome and transcriptional regulation, and multiomic approaches combining both scATAC and scRNA read-outs from the same cell are currently on the rise.

**Figure 1:**
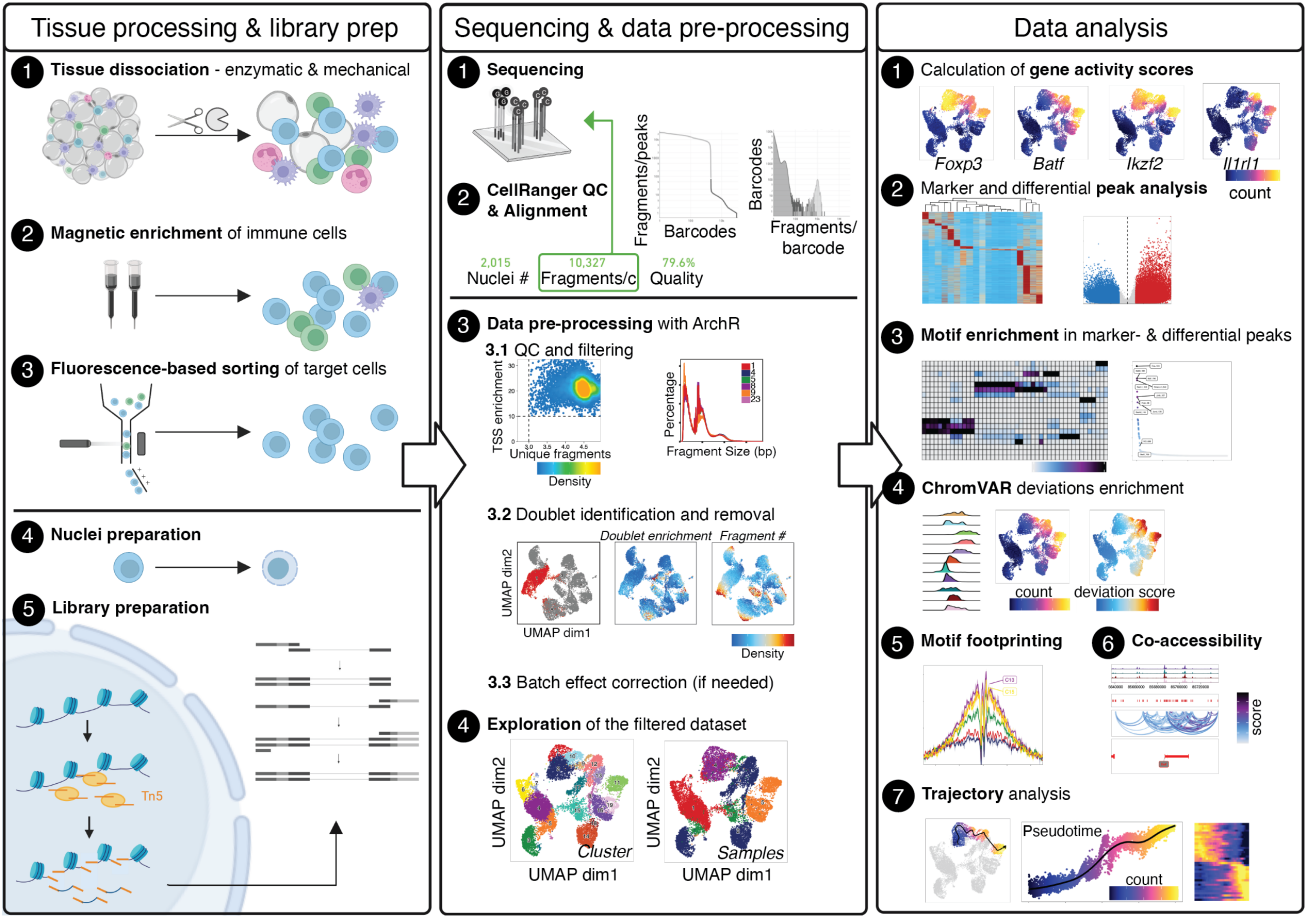
Graphical Abstract describing the whole value chain from tissue processing and scATAC-seq library prep through sequencing and data pre-processing to the analysis of the final, filtered dataset. The Left panel describes tissue processing and library prep: Tissue is enzymatically and mechanically digested (1) and cells are magnetically enriched for target cells (2) to make cell sorting (3) more efficient. After obtaining a pure target cell population (3), cells are made permeable for the Tn5 transposase during nuclei preparation (4), followed by incubation with the Tn5 transposase and library preparation (5). The Middle panel describes sequencing (1) and alignment of fragments, as well as quality control using CellRangerATAC (2). Depending the number of fragments per cell, samples can be sequenced further to yield the desired sequencing depth. Using fragments.tsv files generated by CellRangerATAC count, data is pre-processed with ArchR (3). Steps include setting cut-offs for TSS enrichment and the number of fragments per cell and visual evaluation of the fragment size distribution (3.1), the calculation of doublet scores and removal of doublets (3.2), and, if necessary, batch effect correction (3.3), yielding the final, filtered dataset (4). The Right panel describes data analysis, comprising the calculation of gene activity scores as a proxy for gene expression (1), marker- and differential analysis on the peak matrix (2) as well as motif enrichment analysis in marker- and differential peaks (3). Motif scores can further be calculated on the single-cell level using ChromVAR (4), motif footprinting can be performed (5), co-accessibility of peaks can be assessed (6), and pseudotime analysis can be performed (7). Created with Biorender using figures and plots generated in this manuscript. .

Although different technologies for performing scATAC-seq have been established, they all require the processing of tissue samples for generating single-cell suspensions, the isolation of target cells, the preparation of nuclei, the deposition of individual nuclei in wells or droplets, library preparation and sequencing, and finally bioinformatic analysis. In this methods article, we provide guidance for all steps that are required to perform scATAC-seq on CD4+ T cells from murine tissues (see also **Figure 1**). First, we will describe wet-lab protocols for isolating T cells from murine tissues such as skin, visceral adipose tissue (VAT), colon, or secondary lymphoid tissues such as the spleen. We will describe processing steps for pre-enrichment and purification of target cells, isolation of nuclei and further processing using commercially available droplet-based microfluidic systems and chemistry. We will provide recommendations for cost-efficient and resource-saving sequencing strategies, accompanied by links for the download of freely accessible example datasets where CD4+ T cells from murine tissues were isolated, processed and sequenced as described (8). Then, we will guide the readers through the bioinformatic processing of samples, from initial quality control steps through data pre-processing to the analysis of the final, filtered dataset. This typically includes the calculation of gene activity scores, peak calling and motif enrichment, footprinting, co-accessibility and trajectory analysis. We will provide a reproducible workflow for recreating our findings that readers can extend and adapt to their needs, and provide advice on typical parametrical and procedural errors that may occur during analysis.

## Methods – experimental procedures

**Figure 2:**
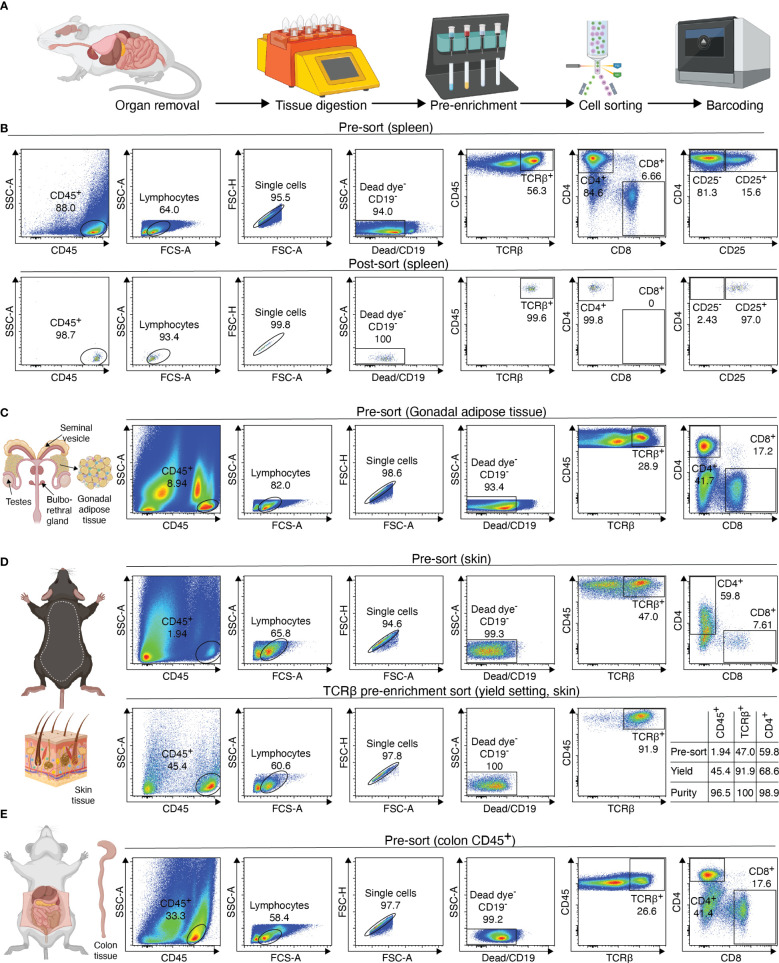
Overview of sample preparation for scATAC-seq of CD4+ T cells from murine tissues. **(A)** Procedural overview. Organs are removed, followed by tissue digest and CD4 T cell enrichment. These are then sorted and processed further for generation of the scATAC-seq library **(B–E)** Flow cytometry plots illustrating the gating scheme to isolate T cells from tissues such as spleen, fat, skin and colon. For skin, a pre-sort enriches target cells, followed by purity sorting. Relative enrichment by each sort in table. Figure elements with Biorender.

The processing of samples for scATAC-seq is the first key step to producing high-quality data. In our experience, low quality cell isolation results in high fragmentation of nuclear DNA, translating into poor library profiles, low sequencing efficiency and bad data quality. Therefore, we will describe key steps for isolating CD4+ T cells from murine peripheral organs compatible with droplet-based microfluidic systems and chemistry from commercial suppliers, with details on organ removal, tissue digestion procedures and enzyme formulations, pre-enrichment of target cells, sorting of viable cells, nuclei isolation, transposition and barcoding (**Figure 2A**). Required equipment for experimental procedure and computing infrastructure is listed in **Tables 1**, **2**, respectively.

**Table 1:** Equipment required for experimental procedures.

Major equipment required to perform experimental procedures	
Name	Manufacturer
Chromium Controller	10X Genomics
Miltenyi GentleMACS	Miltenyi Biotec
High-speed cell sorting system	BD Biosciences or other
Tapestation or Bioanalyzer	Various
Nextseq 500/550	Illumina

**Table 2:** Equipment required for data analysis.

Required computing infrastructure	
Tool	Requirements/Recommendations
Cell Ranger ATAC (v2.0.0)	8-core Intel or AMD processor (24 cores recommended) 64GB RAM (160GB recommended) 10-100GB free disk space per sample (depending on various factors including sequencing depth, sequencing strategy, number of nuclei sequenced) 64-bit CentOS/RedHat 7.0 or Ubuntu 14.04
R/Analysis with and ArchR	Processor 64-bit processor with x86-compatible architecture (such as AMD64, Intel 64, x86-64, IA-32e, EM64T, or x64 chips) 1 GB free disk space 8 GB RAM (32 GB recommended)

### Isolation of T cells from murine spleen

To isolate T cells from murine secondary lymphoid tissues such as spleen, the tissue is harvested, placed in FACS buffer (**Table 3**) and stored at 4°C until use. Then, the spleen is placed on a 100 µM filter unit and is mechanically dissociated using a plunger or forceps. Following centrifugation (2 min, 1000g, 4°C), red blood cells are lysed using a commercially available ACK lysis buffer (e.g. Thermo Fisher *#*A1049201). The cell suspension is filtered using a 70 µm strainer, resuspended in 500 µl FACS buffer, and cells are counted.

**Table 3:** FACS buffer.

Formulation for FACS buffer		
Ingredient	Manufacturer	Final concentration
Phosphate-buffer saline 10X	Gibco #10010023 or other	1X
FCS 100%	Sigma #F7524 or other	2%
Deionized water	NA	Up to final volume

Afterwards, we add Fc blocking reagent (Miltenyi Biotec #130-092-575) to prevent unspecific binding of antibodies and beads, followed by specific labeling using 1 µg PE-conjugated anti-mouse CD4 (Clone RM4-5, Biolegend #100512) or 1 µg PE-conjugated anti-mouse CD25 (Clone PC61, Biolegend # 102008) antibodies in 500 µl and stain for 20 min at 4°C. After staining, cells are centrifuged (2 min, 1000g, 4°C), washed using 1000 µl of FACS buffer, and resuspended in MACS buffer (**Table 4**). In the next step, target cells are bound by anti-PE ultrapure microbeads (Miltenyi Biotec #130-105-639) for 20 min at 4°C, followed again by two centrifugation (2 min, 1000g, 4°C) and washing steps using 1000 µl of FACS buffer. Finally, samples are re-suspended in 500 µl MACS buffer. A 70µl filter unit is placed on an equilibrated MACS column (we recommend working at 4°C to prevent cellular degradation) and the sample is loaded. The column is washed twice with 5 ml MACS buffer.

**Table 4:** MACS buffer.

Formulation for MACS buffer		
Ingredient	Manufacturer	Final concentration
Phosphate-buffer saline 10X	Gibco #10010023 or other	1X
Bovine Serum Albumin 100%	Sigma #A4503 or other	0,5% (w/v)
Ethylenediaminetetraacetic acid	ThermoFisher #15575020	1 mM
Deionized water	NA	Up to final volume

Afterwards, the sample is eluted in 500 µL FACS buffer and stained using fluorescence-labelled antibodies. We recommend a gating strategy where CD4 or CD25 T cells are enriched to high purity, and dead cells, unwanted cell types and doublets are excluded (**Figure 2B**, upper panel). A small part of the sorted population (target cells) can then be re-analyzed before downstream processing to determine post-sort purity and viability (**Figure 2B**, lower panel). If the quality criteria are met, the sample can be subjected to nuclei preparation and further sample processing, as described later. For troubleshooting and recommendations see **Box 1**.

Box 1 **Troubleshooting and Recommendations.**

**Table:** 

Troubleshooting and Recommendations	
Description	Solution
Low cell viability	Analyze buffer ingredients, optimize erythrocyte lysis procedure, keep time spent on isolation of cells as short as possible, work at 4°C
Erythrocyte contamination	Optimize ACK lysis procedure
Low purity of CD4 or CD25 T cells	Use Fc blocking reagent, work at 4°C
Isolation of T cells from spleen vs blood	A higher number of peripheral T cells can be isolated from the spleen as compared to the blood of mice. Also taking into consideration the loss of cells during nuclei preparationaration for scATAC-seq, it is advisable to isolate peripheral T cells from the spleen instead of the blood.
Doublet exclusion during sort	When sorting cells from tissues, naturally occurring cell doublets (biological interaction between T cells and other cell types) can be identified by including markers for these cell types in the sort panel. In our hands, T cell – APC pairs, if not excluded during sorting, are not separated by nuclei preparation and can be detected in subsequent data analysis. This is true for cells isolated from all tissues.

### Isolation of T cells from murine adipose tissue

To isolate T cells from VAT tissue, gonadal fat pads of male mice are excised and placed in either a 50 ml conical tube or a GentleMACS tube (Miltenyi Biotec #130-096-334) containing VAT digestion buffer (**Table 5**). The VAT buffer recipe contains a collagenase subtype to digest the extracellular matrix, DNAse to prevent DNA released from dying cells clogging filters, and BSA to prevent unspecific digestion of cell surface epitopes.

**Table 5:** VAT digestion buffer.

Formulation for VAT digestion buffer		
Ingredient	Manufacturer	Final concentration
DMEM media	Gibco #41965	1X
Collagenase Type II	Sigma #C6885	1 mg/ml
Bovine Serum Albumin	Sigma #A4503	20 mg/ml
DNAse I	Roche #11284932001	20 µg/ml

To support the digestion process, the gonadal fat depots are cut into small pieces using (sharp) scissors and digested for 45 minutes at 37°C. Ideally, the sample should be rotated (e.g. using a rotating device in an incubator), placed in an orbitally shaking waterbath, or stirred and heated automatically using a GentleMACS Dissociator (program: 37C_mr_ATDK_1). Then, the sample is incubated with 10 ml of 2 mM EDTA-PBS for 2 minutes, followed by a centrifugation (5 min, 500g, 20°C). The sample is resuspended in 1000 µl FACS buffer and transferred to a 1.5 ml tube through a 100 µm filter unit. Then, the sample is centrifuged again (2 min, 1000g, 4°C), resuspended in 1000 µl FACS buffer and filtered into a new tube using a 70 µm filter unit. The sample can now be stained for sorting, with an example shown in **Figure 2C**. For troubleshooting and recommendations see **Box 2**.

Box 2 **Troubleshooting and Recommendations.**

**Table:** 

Troubleshooting and Recommendations	
Description	Solution
No gonadal fat depots	Gonadal fat depots are only present in male mice. Younger animals, starving or sick animals have small or no depots.
Erythrocyte contamination	Add ACK lysis step to procedure
Low purity of CD4 or CD25 T cells	Use Fc blocking reagent, work at 4°C
No expression of CD4 or CD8 on T cells	Optimize processing time and amount of collagenase enzymes

### Isolation of T cells from murine skin tissue

To isolate T cells from skin tissue, hair has to be removed from the back of the animal with an electric shaver and depilatory cream. The cream is applied for 2 minutes, followed by vigorous washing using tap water to remove hair. It is important that excess hair is completely removed to avoid complications during downstream filtration steps. After cleaning, the skin is separated from the dorsal surface, cut into small pieces, and transferred to a GentleMACS tube (Miltenyi Biotec #130-096-334) containing 10ml of skin digestion buffer (**Table 6**).

**Table 6:** Skin digestion buffer.

Formulation for skin digestion buffer		
Ingredient	Manufacturer	Final concentration
DMEM media	Gibco #41965	1X
Collagenase Type II	Sigma #C6885	4 mg/ml
Bovine Serum Albumin	Sigma #A4503	20 mg/ml
DNAse I	Roche #11284932001	20 µg/ml

Then, the sample is digested using the GentleMACS Dissociator (program: 37_C_Multi_H) or via orbital shaking in a preheated waterbath (37°C). After 90 minutes of digestion or completion of the GentleMACS program, the single-cell suspension can be cut again, centrifuged (10 min, 400g, 4°C), resuspended in 5000 µl FACS buffer and transferred to a 15 ml tube through a 100 µm filter unit. Then, the sample is centrifuged again (2 min, 1000g, 4°C), resuspended in 1000 µl FACS buffer and filtered into a new 1.5 ml tube using a 70 µm filter unit. The sample can now be stained for sorting, with an example shown in **Figure 2D**. To increase sort efficiency, it might be beneficial to first enrich for CD45^+^ immune cells (yield sort) by sorting target cells into FACS buffer, followed by a second purity sort (4-way purity sort) for target cells. For troubleshooting and recommendations see **Box 3**.

Box 3 **Troubleshooting and Recommendations.**

**Table:** 

Troubleshooting and Recommendations	
Description	Solution
Clogging caused by hair	Additional filter steps after skin digestion get rid of hair and avoid clogging. Repeat hair removal if patches of hair remain.

### Isolation of T cells from murine colon

To isolate T cells from colon tissue, the colon is mechanically separated from small intestine and placed in FACS buffer. Remaining fat and mesenteric lymph nodes are removed. The colon is opened longitudinally, cleared of feces, and transferred to a new tube with 10 ml of colon pre-digestion buffer (**Table 7**). The colon is incubated for 15 min on a bacterial shaker at 225 rpm and 37°C, followed by 30sec of vortex. The solution is passed through a 100 µm filter unit, where the colon remains in the filter and is transferred to a new tube with 10 ml of fresh colon pre-digestion buffer. The flowthrough is discarded and contains epithelial cells, while the lamina propria remains on the filter unit. The colon is incubated again for 15 min on a bacterial shaker at 225 rpm and 37°C, followed by 30sec of vortex and filtration.

**Table 7:** Colon pre-digestion buffer.

Formulation for colon pre-digestion buffer		
Ingredient	Manufacturer	Final concentration
Hank’s Balanced Salt Solution	ThermoFisher #14175095	1X
Ethylenediaminetetraacetic acid	ThermoFisher #15575020	4 mM

The colon pieces are transferred to a 50ml tube containing 10ml of colon digestion buffer (**Table 8**), and scissors are used to cut the colon into small pieces. Digestion is performed in a bacterial shaker for 15 min at 37°C and 225 rpm.

**Table 8:** Colon digestion buffer.

Formulation for colon digestion buffer		
Ingredient	Manufacturer	Final concentration
DMEM media	Gibco #41965	1X
Collagenase Type V	Sigma #C9263	0,85 mg/ml
Collagenase Type D	Roche #11088882001	1,25 mg/ml
DNAse I	Roche #11284932001	20 µg/ml
Dispase	Gibco #17105	1 mg/ml

Upon completion of digestion, the colon can be cut again to increase yield. The cell suspension is now centrifuged (10 min, 400g, 4°C) and resuspended in RPMI media, followed by two additional filtration steps with 10ml of RPMI. The sample can then be resuspended in FACS buffer and either pre-enriched (recommended) or stained for sorting. For troubleshooting and recommendations see **Box 4**.

Box 4 **Troubleshooting and Recommendations.**

**Table:** 

Troubleshooting and Recommendations	
Description	Solution
Fatty cell pellet after digestion	After digestion the cell pellet can contain a lot of fat. If so, add an additional filter step with a 70 µm filter unit.
Clogging during cell sorting	For cell sorting samples should be filtered again immediately before acquisition and cooled at 4°C to avoid clogging.

### Preparation of nuclei and library for scATAC-seq

Cells have been sorted in FACS buffer and stored at 4°C until use. In our experience, it is important to process the samples quickly after sorting to decrease the overall fragmentation of the chromatin. Therefore, shortly after sorting, cells are pelleted by centrifugation (5min, 300g, 4°C). Supernatant is removed and cells are resuspended in 100 μl 0.04%BSA-PBS buffer. Cells are centrifuged again (5min, 300g, 4°C) and supernatant is removed completely. Then, 45 μl chilled lysis buffer (**Table 9**) is added, and lysis occurs for 2 min at 4°C.

**Table 9:** Lysis buffer.

Formulation for nuclei preparation lysis buffer		
Ingredient	Manufacturer	Final concentration
Nuclease-free water	Invitrogen	1X
TRIS-HCL pH7.4	Sigma	10mM
NaCL	Sigma	10mM
MgCl2	Sigma	3mM
Tween-20	Biorad	0.1%
NP-40	Sigma	0.1%
Digitonin	Invitrogen	0.01%
Bovine Serum Albumin	Sigma	1%

After precisely 2 min, 50 µl washing buffer (**Table 10**) is added and the sample is centrifuged (5min, 300g, 4°C).

**Table 10:** Washing buffer.

Formulation for nuclei preparation washing buffer		
Ingredient	Manufacturer	Final concentration
Nuclease-free water	Invitrogen	1X
TRIS-HCL pH7.4	Sigma	10mM
NaCL	Sigma	10mM
MgCl2	Sigma	3mM
Tween-20	Biorad	0.1%
Bovine Serum Albumin	Sigma	1%

**Figure 3:**
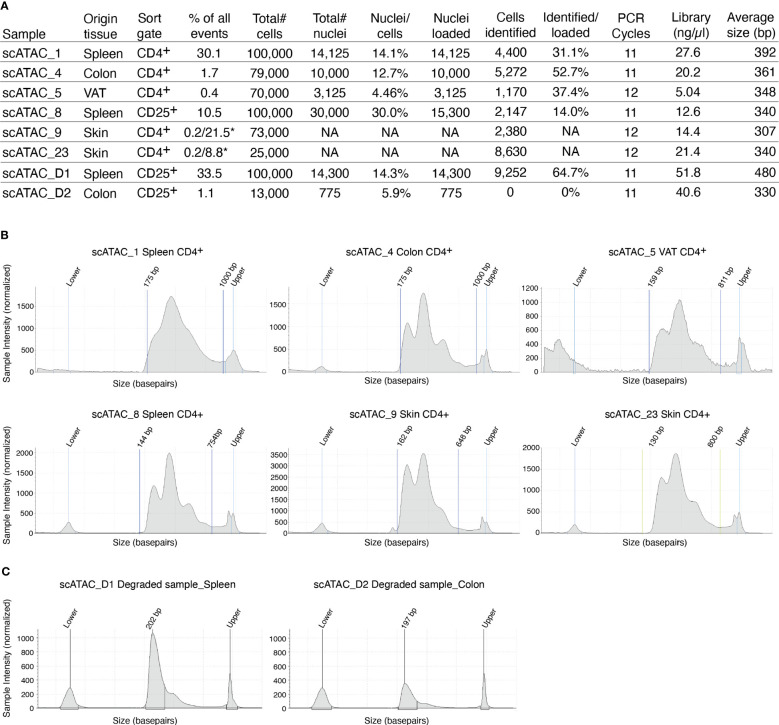
Overview of recovery and typical profiles for scATAC-seq libraries. **(A)** Tabular overview of parameters in scATAC-seq experiments. The percentage of all events indicates the total frequency of target cells (either CD4+ or CD25+ T cells) in all events from the sample. In skin samples, we used a double-sort approach with a yield sort followed by a purity sort, as described earlier and indicated with an *. **(B)** Examples for library size profiles for samples with a good library profile listed in **(A)**. **(C)** Examples for library size profiles of low-quality samples (faulty transposition or strongly degenerated DNA). Profiles were generated using a Tapestation with a high sensitivity D1000 Screentape.

**Figure 4:**
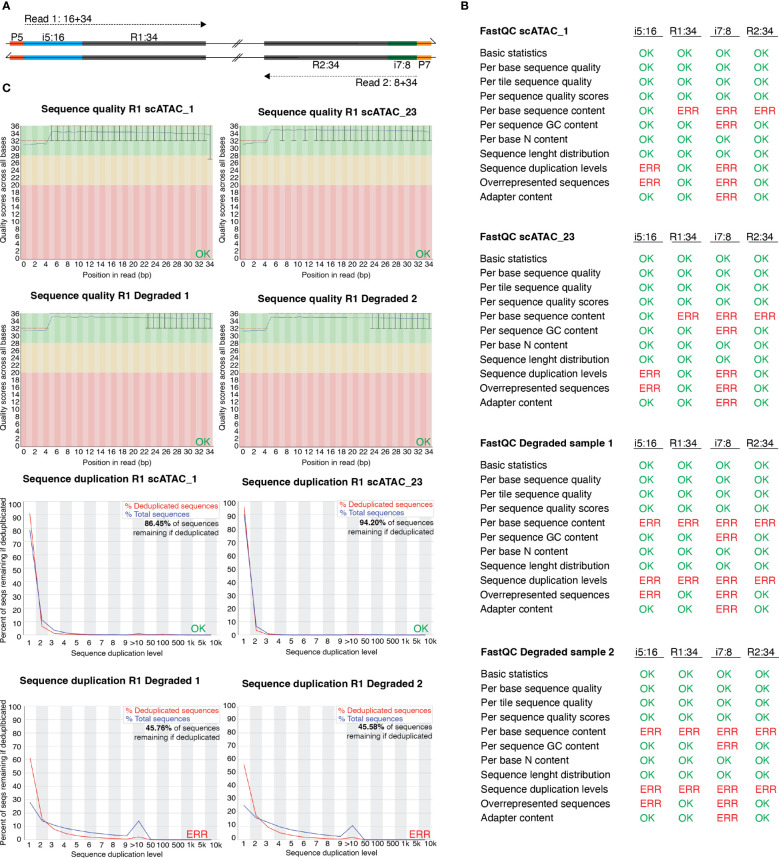
FastQC profiles of good-quality and of degenerated scATAC-seq libraries sequenced using PE-34-8-16-34 strategy. **(A)** Overview of sequencing strategy using a PE-34-8-16-34 approach. **(B)** Statistics of FastQC run on scATAC_1 (MD_1_4_run_1_MD_scATAC_1_S1_ L001_I1_001 (i7:8), …R1_001 (R1:34), …R2_001 (i5:16), …R3_001 (R2:34)), scATAC_23 and the degraded samples scATAC_D1 and scATAC_D2. Errors listed here as reported in FastQC documentation. **(C)** Sequence quality and sequence duplication overview of R1:34 in good-quality samples scATAC1 and scATAC23, and degraded samples scATAC_D1 and scATAC_D2. Produced by FastQC (version 0.11.9).

The supernatant is removed and 45µl of chilled diluted nuclei buffer (10X Genomics #2000207) is added. The sample is centrifuged again (5min, 300g, 4°C) and resuspended in 7 µl chilled diluted nuclei buffer (10X Genomics). At this point, 1 µl of nuclei can be counted using acridine orange/propidium iodide. The nuclei recovery is listed in **Figure 3A** and ranges from 30.0% (spleen CD25+) to 4.5% (VAT CD4+), with 12.7% for colon CD4+ and 14.1% for spleen CD4+. From the nuclei suspension, 5 µl are used in the transposition reaction (Single-cell ATAC Gel Beads V1.0 or V1.1 and reagents, 10X Genomics #1000175) for one hour at 37°C. Samples are supplemented with master mix and beads, loaded on a 10X Chromium Next GEM Chip H (10X Genomics #1000161) and processed on the 10X Chromium Controller (10X Genomics #120212), followed by library preparation according to the manufacturer’s protocol. GEM incubation was performed with 11-12 cycles of PCR based on the number of nuclei in the transposition reaction. As listed in **Figure 3A**, the number of PCR cycles translates directly into the concentration of the library. Upon completion of library preparation, the fragment length composition is usually evaluated using electrophoretic separation of the sample. In **Figure 3B**, examples for library profiles from scATAC-seq data of primary murine CD4+ T cells from spleen and tissues are shown. The fragment size distribution of a high-quality sample should show nucleosomal periodicity, with fragment lengths being enriched in 150bp-steps, which is the circumference of one nucleosome. If nucleosomal periodicity is lost, this can be an indication of degenerated chromatin structure. To illustrate this, we included a sample where either the transposase enzyme was inactive or the DNA itself was highly degraded, leading to a poor library profile (**Figure 3C**). Even so, sequencing this sample will generate reads that per se are of good quality, yet limited in their usefulness, as the library will be of low complexity (**Figure 4**). In addition, we included a low-quality sample with good library profile (Skin CD4+) in this comparison, and although the library profile showed periodicity, the data quality was not sufficient for further analysis. Therefore, library profiles only indicate that the procedure itself has been completed and the DNA was intact, but does not guarantee that all libraries will yield results that can be analyzed and interpreted. On the other hand, if the library is severely compromised (e.g. no periodicity at all), we can anticipate that no meaningful data can be extracted from such samples. For troubleshooting and recommendations see **Box 5**.

Box 5 **Troubleshooting and Recommendations.**

**Table:** 

Troubleshooting and Recommendations	
Description	Solution
Quality of nuclei	After nuclei preparation, about 5% viable cells should be remaining. This indicates, that the lysis was not too harsh and that nuclei are intact. If this is not the case, the lysis time can be reduced.
Variability nuclei/cells	The number of nuclei that are isolated from a certain number of cells is dependent on a variety of factors, including the tissue of origin (harshness of the digestion protocol), the % of all events (sort time), sort efficiency, the flow rate (pressure), the time window between sorting and the nuclei preparation, technical variability during processing of nuclei
Variability identified cells/loaded nuclei	The percentage of cells identified per loaded nuclei is dependent on several factors including the quality of nuclei loaded (intact vs disrupted nuclei) the number of nuclei loaded (multiplets), and the precision of nuclei counting, and usually ranges between 20% and 50%.
Processing of human samples	The isolation of CD4+ T cells from human tissues requires different dissociation protocols, however the nuclei preparationaration as well as scATAC-seq library preparation is identical.

## Methods – sequencing and QC strategy for scATAC-seq libraries

In **Figure 3A**, we listed the total number of nuclei loaded onto the microfluidics systems and the total number of nuclei that were later identified as cellular event. The recovery rates ranged from 14.0% for spleen CD25+ to 52.7% for colon CD4+, with a mean recovery of 33.8%. Therefore, we can roughly estimate the number of nuclei that will be analyzed as about 1/3 of the number of murine tissue T cell nuclei in our sample. Still, the number of identified nuclei varies, which makes sequencing in an all-in-one effort rather risky – while, on the one hand, some samples can be “over-sequenced”, resulting in high numbers of fragments per cell and good coverage, other samples can be “under-sequenced”, resulting in low numbers of fragments per cell and rather poor coverage. An uneven number of fragments per cell across different samples calls for artificial down-sampling of samples with higher sequencing depth, and therefore the removal of perfectly good sequencing reads to a level comparable with the sample of the lowest sequencing depth. Down-sampling can be achieved by subsetting the *fragments.tsv* file of the sample with higher sequencing depth in a way that the median unique fragments per cell equals the median unique fragments per cell of the sample with lower sequencing depth (in analogy to the depth normalization function of *cellranger atac aggr*). If there is high variation in the number of fragments per sample, this can result in a loss of many reads and therefore additional cost. Therefore, we recommend an alternating sequencing and QC strategy, where we sequence only 10% of the required reads using a custom protocol for the NextSeq 500/550 sequencer, followed by estimation of the total number of nuclei, the average fragments per nucleus, general QC parameters (see later), and sequencing saturation using Cell Ranger ATAC (10X Genomics Cell Ranger ATAC 2.0.0). We can then use these parameters to plan the sequencing effort and estimate the fraction of each sample in our sequencing pool (if all samples are sequenced together). This reduces the cost, allows for the detection and removal of low-quality samples, and increases the overall comparability of the datasets. In our laboratory, we sequence 10X scATAC-seq libraries using a paired-end run with 34-8-16-34 sequencing strategy with a 75-cycle high-output cartridge on a NextSeq 500/550. In a typical run, Read 1 identifies the i5 index (cell barcode) with 16 nucleotides and reads 34 nucleotides of the fragment. On the reverse strand, primer P7 initiates the i7 read (sample index) with 8 nucleotides and reads 34 nucleotides of the fragment (**Figure 4A**).

### Quality control of sequencing output files using FastQC

Running aforementioned libraries on an Illumina sequencing machine generates binary base call (BCL) files, from which fastq files can be generated using Illumina bcl2fastq (**Box 6**). *bcl2fastq* takes as input a sample sheet (see 10X Genomics scATAC-seq documentation) stating sample indices present in the loaded library, demultiplexes Illumina BCL files accordingly, and creates an output containing fastq files for each sample.

To investigate whether we can estimate library quality, we ran FastQC (9) on all L001 files generated from four libraries: the “good-quality” libraries scATAC_1 and scATAC_23, which showed periodical profiles upon electrophoretic separation (**Figure 3B**), and the degenerated samples scATAC_D1 and scATAC_D2, which showed degradation already in the library profile (**Figure 3C**). As expected, the overall run quality reports such as per base or per tile sequence quality or per sequence quality scores did not vary between libraries (**Figure 4B**), and can also be visualized (**Figure 4C**, top). In general, QC run on the indices generates errors in duplication rate and overrepresented sequences, which can be expected. In contrast to this, the degenerated libraries had high sequence duplication levels also in their long reads R1:34 and R2:34, which indicates low library complexity, leading to uninformative samples. This can also be seen when plotting sequence duplication levels (**Figure 4C**, bottom). Data from the libraries scATAC_D1 and scATAC_D2 did not yield any biologically meaningful information, and the sequencing was stopped after results from FastQC and Cell Ranger ATAC identified these problems.

Box 6 **bcl2fastq.**

**Table:** 

Terminal input to run *bcl2fastq*	
$ bcl2fastq --use-bases-mask=Y34,I8,Y16,Y34 \ --create-fastq-for-index-reads \ --minimum-trimmed-read-length=8 \ --mask-short-adapter-reads=8 \ --ignore-missing-positions \ --ignore-missing-controls \ --ignore-missing-filter \ --ignore-missing-bcls \ -r 6 -w 6 \ -R /media/raw_data/NextSeq/*name_of_the_run* \ --output-dir=/media/raw_data/NextSeq/*name_of_the_run*/fastq \ --sample-sheet=/media/raw_data/NextSeq/*name_of_the_run*/SampleSheet.csv \ --no-lane-splitting

### Running *Cell Ranger ATAC count* to estimate re-sequencing needs of libraries

As mentioned before, we sequence a small amount of the library (typically 10%) and run FastQC and *Cell Ranger ATAC count* to get a first glimpse of the library quality, the number of cells and the number of fragments per cell, sequencing saturation and other parameters. *Cell Ranger ATAC count* (**Box 7**) takes fastq files as input and aligns fragments to the specified reference genome (in our case we chose the murine reference mm10, for human data the human reference genome GRCh38 is available). Amongst other outputs, a summary html file is created. Based on the number of fragments per cell, the number of sequenced read pairs, and the sequencing saturation, an estimate can be made of how much deeper the sample has to be sequenced. Upon re-sequencing, *Cell Ranger ATAC count* can be performed on fastq files from both the first and the second sequencing run together, and appropriate sequencing depth and quality of the sample can be confirmed.

Box 7 **Cell Ranger ATAC count.**

**Table:** 

Terminal input to run *Cell Ranger ATAC count*	
*# download the appropriate reference data from* *# https://support.10xgenomics.com/single-cell-atac/software/downloads/latest* *# move to the directory you want the output to be written to and prepend cellranger-atac* $ cd ~/directory $ export PATH=./path/to/cellranger-atac-2.0.0:$PATH *#run cellranger-atac count for several files (fastq files from multiple runs)* $ for x in samplename1 samplename2 samplename3; do cellranger-atac count \ --localcores=10 \ --id=name_your_sample_"$x" \ --reference=./path/to/reference/data\ *#download see 10X documentation* --fastqs=./path/to/fastq/files,./path/to/more/fastq/files \ --sample="$x"; done

### Combining sequencing files and running *Cell Ranger ATAC count* to create output files for downstream analysis with *ArchR*

Once the desired sequencing depth is reached, *Cell Ranger ATAC count* is run with fastq files from all sequencing runs of a certain sample as input (see **Box 7**). Upon alignment to the reference genome, a tabix sorted text file containing fragment start- and end position and the corresponding cell barcodes is created, which serves as input for the downstream processing with ArchR. The *fragments.tsv.gz* file (~2GB for 5.000 cells with a read depth of 10.000 reads/cell) only contains fragments which have passed the following QC criteria: The fragment must be mapped with a MAPQ > 30 on both reads, it must be non-mitochondrial, not chimerically mapped, and must map to a primary contig. Fragments that share the same cell barcode, start- and end position are further recognized as duplicates generated from the same template during amplification, and one representative fragment is kept for each group of duplicates.

## Methods – data pre-processing with ArchR

In this paragraph, we describe the pre-processing of scATAC-seq data using ArchR ( (10), v1.0.1), including QC and filtering, dimensionality reduction, removal of doublets, evaluation of batch effect correction, which generates the final filtered dataset for analysis. For data pre-processing and analysis with ArchR we provide the code in a GitHub repository (https://github.com/imbeimainz/scATACseq_TissueTcells) as well as an html file containing all code and output from the analysis of our test dataset (https://zenodo.org/record/8160122), which we refer to in the corresponding paragraphs.

### Creating the Count Matrix from Cell Ranger ATAC output

scATAC-seq data analysis is performed on a count matrix, containing the Tn5 insertion counts per genomic region per cell. As for any specific region we either get insertions (open chromatin) or no insertion (closed chromatin or no transposition event), the scATAC-seq count matrix is very sparse. In ArchR, the count matrix can be constructed from the *fragments.tsv* file output by Cell Ranger ATAC, which is a tabix-sorted text file containing chromosome, beginning- and end position of each sequenced fragment along with the corresponding cell barcode. For the count matrix, the genome is subdivided into 500bp-tiles, and the insertion counts are listed per cell per tile.

In ArchR, an *arrow file* is created from the *fragments.tsv* file of each sample, to which metadata and sequence-derived data like the tile matrix are added (**Box 8**). The arrow file is a HDF5 format file to which layers of additional information (e.g. gene score matrix, peak count matrix etc.) can be appended later on. For analysis, arrow files are combined into an *ArchRProject* (**Box 9**). Having the arrow files as HDF5 makes it possible to access the data on-disk rather than having to load it into memory, which would be much more resource-consuming. See sections “2 Create ArrowFiles” and “3 Create ArchRProject”. It is possible at any point during analysis to convert the ArchRProject to a Seurat object using the ArchRtoSignac package (https://github.com/swaruplabUCI/ArchRtoSignac), favoring the interoperability among existing workflows. Similarly, it is possible to convert such objects into SingleCellExperiment objects, widely adopted throughout the Bioconductor ecosystem of packages, where users can e.g. interactively explore their data with iSEE (11) or other software.

Box 8 **Creating arrow files.**

**Table:** 

R code for creating arrow files	
*# Read in fragments files* samples = paste0("MD_scATAC_", c(1,4,5,8,9)) inputFiles = file.path("data", samples, "fragments.tsv.gz") names(inputFiles) = paste0("scATAC_", c(1,4,5,8,9)) *# Create arrow files* *# Evaluate different thresholds depending on your data:* *# - minTSS: Start with 0 to see all cells, afterwards evaluate which threshold* *# works for all of the samples* *# - minFrags: Recommended to set >= 1000, otherwise the analysis might not be* *# robust enough* *# Check different parameters to set with ?createArrowFiles* ArrowFiles = createArrowFiles( inputFiles = inputFiles, sampleNames = names(inputFiles), minTSS = 0, minFrags = 1000, addTileMat = TRUE, addGeneScoreMat = TRUE, force = TRUE )

Box 9 **Creating the ArchRProject.**

**Table:** 

R code for creating the *ArchRProject*	
*# Define arrow files* ArrowFiles = paste0(("scATAC", c(1,4,5,8,9), ".arrow") *# Create ArchRProject* *# It is recommended to set copyArrows = TRUE to maintain an unaltered copy for* *# later usage.* proj = ArchRProject( ArrowFiles = ArrowFiles, copyArrows = TRUE )

### Per-cell QC and filtering for high-quality cells

**Figure 5:**
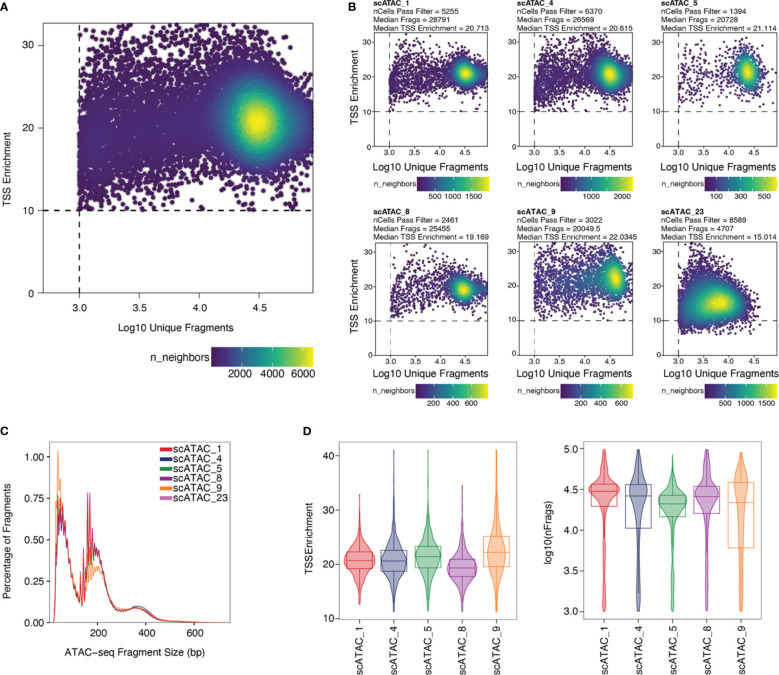
Unique fragments vs TSS enrichment. **(A)** TSS enrichment vs log10(unique fragments) of the ArchRProject displayed as scatter plots. Each datapoint is colored by the number of neighboring datapoints. **(B)** TSS enrichment vs log10(unique fragments) of each individual sample in the dataset. Samples scATAC_1, scATAC_4, scATAC_5, scATAC_8, and scATAC_9 show comparable TSS enrichment and unique fragments. Sample scATAC_23 has both a lower mean TSS enrichment, i.e. lower quality, and a lower mean number of unique fragments and was therefore excluded from analysis. **(C)** Fragment size distribution displaying nucleosomal periodicity of 150bp. **(D)** Violin plots of TSS Enrichment and the number of fragments per cell for all samples after setting the cut-offs for TSS Enrichment and the number of unique fragments per cell.

Stringent filtering for high-quality cells is required prior to analysis. Quality parameters implemented in ArchR’s quality control are fragment size distribution, number of unique nuclear fragments, and signal-to-background ratio. The fragment size distribution of a high-quality sample should show nucleosomal periodicity, with fragment lengths being enriched in 150bp-steps, which is the circumference of one nucleosome (**Figure 5C**). If nucleosomal periodicity is lost, this can be an indication of degenerated chromatin structure. A certain number of unique nuclear fragments per cell is required for a robust analysis, therefore a cut-off can be set accordingly. In our analysis, we discarded cells with less than 1000 unique fragments per cell. Non-nuclear, i.e. mitochondrial, fragments are enriched in dead or dying cells. Those fragments are identified by Cell Ranger ATAC and are excluded from the *fragments.tsv* file that serves as an input for ArchR, as are chimerically mapped reads and reads not mapping to a primary contig. The signal-to-background ratio can be quantified via the enrichment of fragments at transcription start sites (TSS) compared to TSS-distal regions. This quality metric is based on the observation that in viable cells, chromatin is more accessible at TSS regions due to the large protein complexes that bind there. Loss of the relative enrichment of fragments at TSS sites again can indicate degeneration of the chromatin structure. In order to choose cut-offs fitting all samples to be included in the analysis, it is advisable to plot the unique nuclear fragments per cell against the TSS enrichment for each sample, and to set the cut-offs accordingly, see section “2 Create Arrow files”, **Box 8**–**11**. TSS enrichment vs unique fragment values of all cellular events in the ArchRProject are displayed as density scatter plots in **Figure 5A**, and of each sample separately in **Figure 5B**. As all samples contain very similar cell types (CD25+ T cells for scATAC_8 and CD4+ T cells for the remaining samples), we expect similar distributions of TSS enrichment. We can observe comparable profiles for the samples scATAC_1, scATAC_4, scATAC_5, scATAC_8, and scATAC_9. In contrast, sample scATAC_23 shows both decreased TSS enrichment and unique fragments per cell (**Figure 5D**). If we combined this sample with the other samples, down-sampling to the median number of fragments per cell of scATAC_23 would be required (for instructions on how downsampling can be achieved see **Box 10**). However, this would remove a lot of information from the other samples. Further, the TSS enrichment, as a proxy for the overall data quality, would still be lower compared to samples scATAC_1-9. Therefore, at this point, the sample scATAC_23 was removed from analysis. As displayed before, this sample could not be distinguished from high-quality samples scATAC_1-9 by fragment size distribution, showing the expected nucleosomal periodicity (**Figure 3B**), or by FastQC (**Figure 4B, C**).

Box 10 **Troubleshooting and Recommendations.**

**Table:** 

Troubleshooting and Recommendations	
Description	Solution
Choose cut-offs	To get an overview of the quality of your data, plot the TSS enrichment against the number of fragments per cell for each of the samples. Choose appropriate cut-offs. Optimally, they should be the same for all samples analyzed together
Differing number of fragments per cell	Sequence libraries with low coverage deeper (unless sequencing saturation is too high already) or down-sample unique fragments per cell of samples which were sequenced too deeply. The latter can be achieved by using *cell ranger aggr* with depth normalization, or by subsetting the *fragments.tsv* file of the sample with higher sequencing depth in a way that the mean number of unique fragments per cell is identical between samples.

Box 11 **Subset the project to cells making the TSS enrichment cut-off.**

**Table:** 

R code for subsetting the project to cells making the TSS enrichment cut-off	
*# Filter for cells passing the TSS enrichent cut-off determined above* proj = proj[proj@cellColData$TSSEnrichment >= 10, ]

### Dimensionality reduction using an iterative LSI approach

With scATAC-seq data, there are several challenges when it comes to dimensionality reduction: Firstly, we have a vast number of features at hand, from which we need to select the ones with a higher degree of variability (i.e. carry the information) within the dataset. Moreover, the transposition events contain the information that this site is accessible, yet it might be difficult to distinguish a non-accessible region (a “biological zero”) from a non-sampled region (a “technical zero”). And finally, the sparsity of the matrix makes many of the commonly used methods for dimensionality reduction, e.g. PCA, not directly applicable to the data at hand.

For scATAC-seq data, latent semantic indexing (LSI) is used for dimensionality reduction, which originally stems from language processing and which was developed especially for sparse data (12). LSI was first used for the analysis of scATAC-seq data by Cusanovich et al. (5), and is performed on the tile matrix as follows: 1) The “term frequency”, i.e. the frequency of accessible tiles, is calculated per single-cell with normalization for sequencing depth; 2) The resulting values are then divided by the “document frequency” (i.e. in how many cells of the dataset a certain tile is accessible) to calculate the term frequency – inverse document frequency (TF-IDF) matrix. TF-IDF penalizes a term that is present in many documents. In scATAC-seq data, chromatin regions that are accessible in many cells and thus do not contribute much to telling cell types apart are penalized, as are regions that are not accessible in any of the cells. 3) Singular value decomposition (SVD) for dimensionality reduction.

Specifically, in ArchR, an iterative LSI approach is implemented (described in (13) in more detail), which initially does an LSI transformation based on the most accessible features, and then performs further iterations based on the most variable features across the clusters computed in the previous iteration. An issue with dimensionality reduction is often that the first LSI component correlates strongly with sequencing depth. This is why e.g. in Signac (14), the first LSI component is dropped. In ArchR, dimensions with a correlation to sequencing depth > 0.75 are excluded automatically. Dimensionality reduction (**Box 12**) is showcased in “5 Dimensionality reduction”, and a varying number of iterations, variable features and the dimensions to use as a means to minimize the influence of technical variability are applied in “5.5 Tweak different parameters of LSI dimensionality reduction”. For troubleshooting and recommendations see **Box 13**.

Alternative approaches to LSI are presented in the results of Chen et al., 2019 (15), adopting e.g. some forms of summarization such as gene activity scores or quantifications into meta-features, followed by steps commonly used in the analysis of scRNA-seq data.

Box 12 **Dimensionality reduction.**

**Table:** 

R code for dimensionality reduction	
*# LSI dimensionality reduction* proj = addIterativeLSI( ArchRProj = proj, useMatrix = "TileMatrix", name = "IterativeLSI", iterations = 2, clusterParams = list( resolution = 0.2, sampleCells = 10000, n.start = 10 ), varFeatures = 25000, dimsToUse = 1:30, force = TRUE )

Box 13 **Troubleshooting and Recommendations.**

**Table:** 

Troubleshooting and Recommendations	
Description	Solution
Batch effects after dimensionality reduction	Increase the number of iterations, decrease the number of variable features, or exclude LSI1
Correlation to sequencing depth	ArchR automatically filters out LSI components with a strong correlation to sequencing depth; however, other technical noise can also strongly influence LSI1
dimsToUse parameter	The number of dimensions used for dimensionality reduction impacts how well subsequent clustering results represent cell type identity. It can therefore be useful to test several dimensionalities

### Clustering using the Louvain or Leiden algorithm

**Figure 6:**
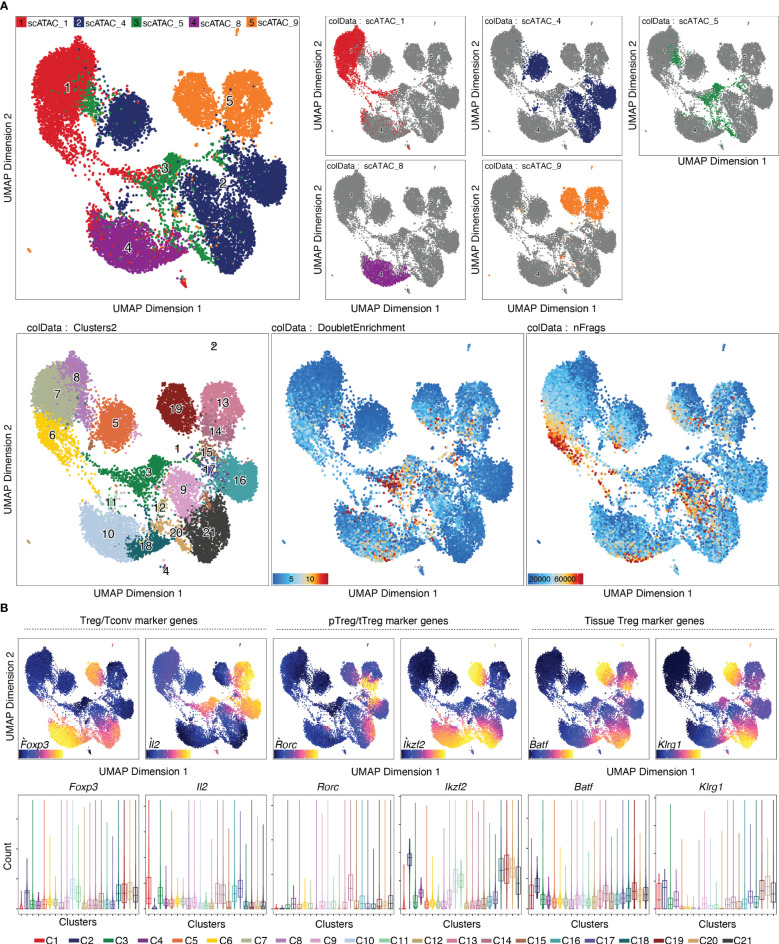
Dimensionality reduction, clustering and visualization in UMAP embedding. **(A)** UMAP colored by samples, with the single samples highlighted (top row), clusters, doublet enrichment and the number of unique fragments per cell (bottom row, left to right). **(B)** Imputed gene scores for a selection of marker genes overlayed on UMAP (top) or as violin plots (bottom).

Per default, ArchR uses the Louvain algorithm (16) for clustering, which is a heuristic graph-based clustering approach. In this approach, a k-nearest neighbor (kNN) graph (17) is constructed, in which each cell is connected to the k nearest cells in Euclidean distance in PCA space. The edge weights are refined based on the Jaccard distance, which evaluates the similarity or overlap of neighboring cells. The cells are then clustered using the Louvain algorithm, which is a heuristic clustering approach used for large datasets, and which performs clustering by optimizing for modularity (method described in (18)). It is also possible to use the Leiden algorithm for clustering, which has been shown to be both faster than the Louvain algorithm and to identify better partitions (19). This can be done by passing *algorithm = 4* to the addClusters() function (**Box 14**). Clustering can thereafter be visualized in a UMAP embedding, as shown in **Figure 6** (see also section “5.2 Visualization in UMAP embedding”, **Box 14**). For troubleshooting and recommendations see **Box 15**.

Box 14 **Clustering and visualization.**

**Table:** 

R code for clustering and visualization as UMAP	
*# Clustering using the Louvain algorithm* *# The Leiden algorithm can be using instead by passing “algorithm = 4”, which is* *# an argument of Seurat’s FindClusters() function, to the addClusters() function* *# (requires the leidenalg Python package)* proj = addClusters( input = proj, reducedDims = "IterativeLSI", method = "Seurat", name = "Clusters", resolution = 0.8, force = TRUE ) *# Visualization of clustering as UMAP* proj = addUMAP( ArchRProj = proj, reducedDims = "IterativeLSI", name = "UMAP", nNeighbors = 30, minDist = 0.5, metric = "cosine", force = TRUE ) *# Color by clusters* p_clusters <- plotEmbedding( ArchRProj = proj, colorBy = "cellColData", name = "Clusters", embedding = "UMAP", size = 0.5 )

Box 15 **Troubleshooting and Recommendations.**

**Table:** 

Troubleshooting and Recommendations	
Description	Solution
Cluster resolution	Always check whether clustering makes sense biologically. It can be helpful to start with a higher clustering resolution and then decrease, to make sure you are not losing any cell populations of interest (overlay gene scores). The package “clustree” can additionally be useful for visualizing how clusters change over increasing resolutions.

### Removal of cell doublets and further filtering steps

In droplet-based single-cell technologies, droplets that received a single barcoded bead but more than one nucleus are referred to as “doublets”, which need to be removed prior to data analysis. To this end, a doublet score can be calculated as in the original ArchR implementation, which works as follows: Synthetic doublets are calculated from the data by combining any combination of two cells, and are projected onto the UMAP space. Their nearest neighbors are identified using the kNN algorithm, and enrichment scores are computed. Enrichment scores can then be overlayed on the UMAP embedding to facilitate pattern recognition across cells.

**Figure 7:**
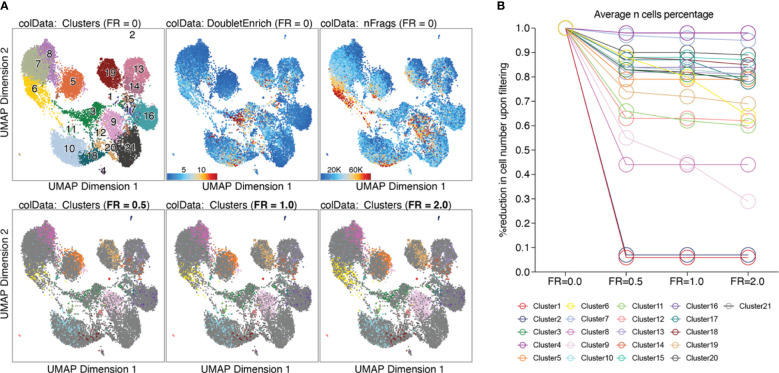
Filtering doublets. **(A)** UMAP colored by clusters, doublet enrichment, and the number of unique fragments per cell (top row). Cells which are filtered out upon applying different filter ratios are highlighted in the respective UMAP (bottom row). **(B)** For each cluster, the reduction in cell number upon applying different filter ratios is plotted.

Based on the calculated doublet score, a filter ratio can be applied to drop the specified percentage of cells with the highest doublet scores. To find an appropriate filter ratio, different considerations can be made: 1) Depending on the number of nuclei loaded on the chip, a certain number of cell multiplets is expected (Chromium Next GEM Single-cell ATAC Reagent Kits v1.1 User Guide CG000209). The filter ratio can be chosen accordingly. 2) As doublets are a mixture of two cells, they can usually be found between two clusters on the UMAP. 3) Doublets are expected to have a rather high number of reads, as they contain reads from two cells. Nevertheless, the number of reads can also be cell type- or quality-dependent. 4) It further makes sense to overlay gene scores onto the UMAP to evaluate whether a cluster has activity in markers from two different cell types, and to make sure you do not remove an entire cell type. It is generally advisable to always check whether the biology makes sense. Different filter ratios can then be applied, and the filter ratio which makes most sense both technically and biologically should be chosen for filtering. Testing of different filter ratios and filtering of doublets is showcased in “6 Filter doublets”, and UMAPs colored by cluster are shown in **Figure 7A** for the filter ratio of 0.5, 1, and 2. **Figure 7B** shows the reduction of cells per cluster upon filtering. Note that filtering out cells using the specified filter ratio removes a certain percentage of cells with the highest doublet scores. The number of cells filtered from each sample therefore depends on the total number of cells in the sample.

Prior to doublet filtering (**Box 16**), cells that are marked by Cell Ranger ATAC as gel bead doublet, barcode multiplet, or low-targeting, should be excluded from the analysis, see section “5.6 Filter out barcodes marked as non-cell by Cell Ranger” and **Box 17**. This information is stored in the *singlecell.csv* file output by Cell Ranger ATAC count, and the *ArchRProject* can be subset to only contain cells that meet these criteria. After doublet filtering, further filtering steps can be performed similarly, e.g. filtering for cells with a certain threshold for mitochondrial reads.

Upon removing cells from the ArchRProject, LSI dimensionality reduction, clustering, and UMAP need to be re-computed. For troubleshooting and recommendations see **Box 18**.

Box 16 **Filtering doublets.**

**Table:** 

R code for filtering doublets	
*# Calculate doublet scores on the ArchRProject* proj = addDoubletScores( input = proj, k = 10, knnMethod = "UMAP", LSIMethod = 1, force = TRUE ) *# After trying out different filter ratios, create new ArchRProject and filter doublets with a filterRatio of 0.5* proj = filterDoublets( proj, filterRatio = 0.5, )

Box 17

**Table:** 

R code for filtering out barcodes marked as non-cell by Cell Ranger ATAC	
singlecell = list() **for** (x **in** c("1","4","5","8","9")){ filename = paste("data/MD_scATAC_",x,"/singlecell.csv",sep = "") data = read.csv(filename) *# To match quality information to cells, we need the barcodes to match the* *# ones in our ArchRProject. For this we:* *# 1) create a vector of "scATAC_x#"* *# 2) add vector as a column to the data* *# 3) create column containing ArchRProj-style barcodes* bc = c(rep(paste("scATAC_",x,"#",sep = ""),nrow(data))) data_barcode = cbind(bc,data) data_fullbc = data_barcode %>% unite("full_barcode", bc:barcode, remove = FALSE, sep = "") singlecell[[x]] = data_fullbc } *# Combine the dataframes* singlecell_fullbc = rbindlist(singlecell, use.names = FALSE, fill = FALSE) *# Extract rownames that are also in the ArchRProject* rownames_archr = rownames(proj@cellColData) subset_singlecell_fullbc = singlecell_fullbc[singlecell_fullbc$full_barcode %**in**% rownames_archr, ] *# Extract is:cell_barcode column from singlecell.csv and give it barcodes as rownames* df_is_cell_barcode = as.data.frame(subset_singlecell_fullbc$is:cell_barcode) rownames(df_is_cell_barcode) = subset_singlecell_fullbc$full_barcode *# Order is_cell_barcode the way the ArchRProject is ordered and create filter* is_cell_barcode = df_is_cell_barcode[order(match(rownames(df_is_cell_barcode), rownames_archr)), ] filter_archr = is_cell_barcode==1 *# Filter out barcodes marked as non-cell by Cell Ranger ATAC* proj = proj[filter_archr, ]

Box 18 **Troubleshooting and Recommendations.**

**Table:** 

Troubleshooting and Recommendations	
Description	Solution
Sample heterogeneity	When calculating the doublet scores, ArchR prints the R^2 of the UMAP projection, which should be above 0.9. If this is not the case, the heterogeneity within the samples is too low to accurately call doublets, as the synthetic doublets would then look too similar to the actual cells the sample contains. In that case, either skip doublet inference or choose knnMethod = “LSI”.
Filter ratio	Test different filter ratios on your dataset, and choose one that makes sense both technically (percentage of multiplets you would expect according to the number of nuclei loaded) and biologically (cell populations according to gene scores).

### Dataset Integration using HARMONY

When samples that are to be analyzed together have a lot of technical variability, sometimes the iterative LSI is not enough to get rid of all non-biological differences. In these cases, a harmonization tool like HARMONY can be employed (20). HARMONY uses a soft k-means clustering algorithm that penalizes clusters that are homogeneous regarding the dataset-origin of the cells they contain, and thus favors the clustering of cells from different datasets. The centroids of these clusters are then used for computing cluster-specific correction factors, which is meant to eliminate dataset-specific differences, while maintaining biological differences (20). Results from batch effect-corrected dataset should, however, be treated with care. Batch effect correction methods like HARMONY, beside the desired effect of reducing the impact of technical variability on the clustering, also affect biological effects. This has been shown to lead to lower reproducibility of cell-type specific markers in batch effect-corrected datasets, although selecting higher cut-offs for effect size and p-value partly mitigates this effect (21).

**Figure 8:**
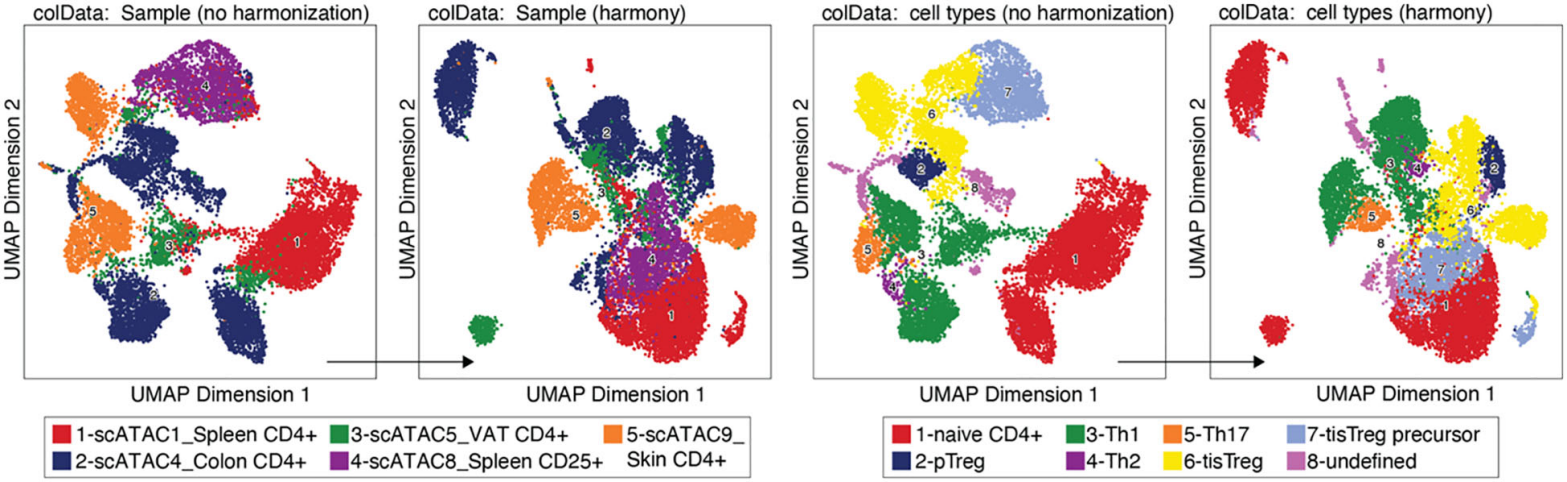
Batch effect correction using HARMONY. UMAP without (left) and with (right) harmonization using HARMONY, colored by sample or tissue type (left panel) and by cell type annotation (right panel).

We performed data integration with HARMONY (**Box 19**), yet we decided not to include this step, since it did not produce the desired effect of the same cell type from different samples clustering together, as shown in **Figure 8**. (see also section “7 Test batch effect correction using HARMONY”). On the contrary, some cell types (e.g. naïve CD4+ T cells or tissue Treg precursor cells) seem to be separated by sample after harmonization. We would like to bring to the attention of the readers that alternative methods exist, such as MNN, Liger, and Conos (22, 23). For troubleshooting and recommendations see **Box 20**.

Box 19 **Batch effect correction using HARMONY.**

**Table:** 

R code for batch effect correction using HARMONY	
*# Create a new ArchRProject with a reducedDims object named “Harmony”* proj_harmonyTest = addHarmony( ArchRProj = proj, reducedDims = "IterativeLSI", name = "Harmony", groupBy = "Sample" )

Box 20 **Troubleshooting and Recommendations.**

**Table:** 

Troubleshooting and Recommendations	
Description	Solution
Recommendation	Only use batch effect correction when necessary.
Technical vs biological variability	If you choose to do batch effect correction, be aware of the fact that while this might reduce the impact of technical variability on the clustering, it might also take away some of the biological effects. The strength of batch effect correction can be influenced by the parameters sigma (width of soft k-means clusters), lambda (ridge regression penalty parameter), and theta (diversity clustering penalty parameter). Treat results from batch effect-corrected datasets with care.
Markers	When extracting cell-type specific markers from batch effect-corrected datasets, make sure to choose an appropriately large cut-off for effect size and p-value.

## Methods – advanced data analysis with *ArchR*

### Cell type annotation

There are several options for cell type annotation: 1) Manual, cluster-based cell type annotation using prior-knowledge marker genes, 2) data-based cell type annotation using cell type annotation tools such as SingleR (24), and 3) Identifying cell types of interest using published signatures for the respective cell type.

#### Manual cell type annotation using gene scores of prior-knowledge marker genes

Based on the accessibility of gene-encoding regions and their regulatory elements, a proxy for gene expression can be estimated. This is done by calculating gene scores. In ArchR, gene scores are calculated as follows: tiles within the gene window of a certain gene are identified, and the ones that overlap with another gene region are excluded. Of the remaining tiles, the distance to the gene is calculated and an exponential weighing function is applied to also take into consideration distal regulatory elements. To address the bias resulting from the fact that large genes tend to have more accessible regions than smaller genes, the latter get larger weights.

**Figure 9:**
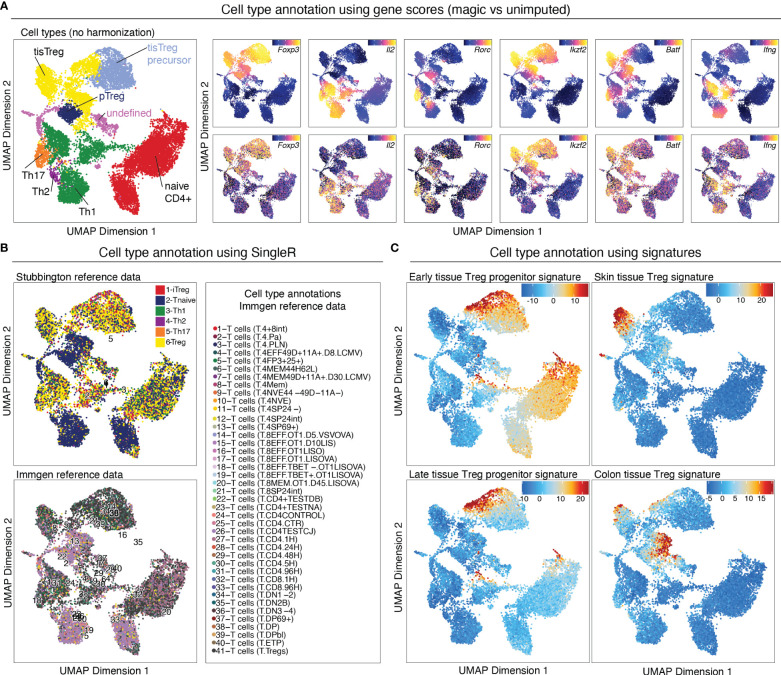
Cell type annotation. **(A)** Manual cluster annotation based on gene scores (left panel). Overlay of gene scores of marker genes with imputation (right panel, top) and without imputation (right panel, bottom) on the UMAP embedding. **(B)** Cell type annotation using SingleR with two different reference datasets. **(C)** Cell type annotation using signatures for specific cell types. Overlay of tissue Treg early progenitor (top left), late progenitor (bottom left), skin tissue Treg (top right), and colon tissue Treg (bottom right) signature z-scores on the UMAP embedding.

Gene scores can be calculated directly during arrow file creation or can be added later. Since we found the gene scores particularly useful during QC and filtering, we generated them directly when creating the *arrow files* by setting the parameter *addGeneScoreMat* to *TRUE*, see section “2 Create ArrowFiles”. Due to the sparsity of scATAC-seq data, gene score plots may appear quite variable. Therefore, imputation using MAGIC (25) can be used to smooth gene scores across nearby cells. Imputed gene scores can then be mapped on the UMAP embedding (**Box 21**). **Figure 9A** demonstrates how imputation facilitates visual interpretation of the data. Cell types of interest can be identified using gene scores of prior-knowledge marker genes in combination with sample information: According to the gene scores of Foxp3 and Batf, clusters C10 and C11 seem to be lymphoid tissue Treg cells, whereas clusters C12, C14, C15 are tissue Treg cells from colon, skin, and VAT, respectively (**Figure 9A**). Manual cell type annotation using gene scores is showcased in “2.1 Manual cluster-based annotation using prior-knowledge marker genes”. For troubleshooting and recommendations see **Box 22**.

#### Reference data-based cell type annotation using SingleR

Cell type annotation can further be performed in a reference data-based manner using SingleR. SingleR was developed for the annotation of scRNA-seq data and can be used with built-in reference datasets, but also accepts custom reference datasets. We annotated our data using the ImmGen database (26) as well as the “Th-Express” mouse CD4+ T cell transcriptome atlas (27). As shown in **Figure 9B**, annotation with both reference datasets identified Treg cells in most of the clusters with an increased Foxp3 gene score. However, additional clusters, which we did not identify as Treg cells using gene score-based annotation, were falsely identified as Treg cell clusters using SingleR. For reference data-based cell type annotation the choice of reference dataset (i.e. how well the cell types match the dataset which is to be annotated) is crucial. It is also important to keep in mind that we are comparing computed gene scores based on chromatin availability with RNA-seq data. Cell type annotation using SingleR is showcased in “2.2 Reference data-based annotation using SingleR”.

Alternatives to the cell type annotation using SingleR exist, such as Seurat’s label transfer approach (28) and scmap (29). Data-based cell type annotation tools are benchmarked in (30, 31). Moreover, if users want to refine the results of such automated annotation tools, manual steps might be required; we refer to the work of Clarke et al. (2021) for additional guidance (32).

#### Identifying cell types of interest using published signatures

For identifying cell types of interest, z-scores for cell type-specific signatures can be calculated on the peak matrix (see section below), and overlayed on the UMAP (**Figure 9C**, **Box 23**). This can be done using the *addDeviationsMatrix* function, which uses functionality from the ChromVAR package ( (33), see below). We calculated z-scores for early- and late tissue Treg progenitors, as well as skin and VAT tissue Treg cell signatures (8) and overlayed them on the UMAP (**Figure 9C**), which confirms the classification we did using gene scores. Cell type annotations based on signature z-scores is showcased in “4.1.2 Calculate signature scores”.

Box 21 **Overlaying gene scores on the UMAP embedding.**

**Table:** 

R code for overlaying gene scores on the UMAP embedding	
*# Overlay gene scores on UMAP embedding of proj, use MAGIC smoothing* *# Define which genes to plot* markerGenes = c("Foxp3","Il2","Rorc","Ikzf2","Batf","Klrg1","Tbx21","Ifng") *# Add impute weights* roj = addImputeWeights(proj) *# Plot* magic_genes = plotEmbedding( ArchRProj = proj, colorBy = "GeneScoreMatrix", name = markerGenes, embedding = "UMAP", plotAs = "points", imputeWeights = getImputeWeights(proj), size = 0.5 )

Box 22 **Troubleshooting and Recommendations.**

**Table:** 

Troubleshooting and Recommendations	
Description	Solution
Gene-dense areas	It is important to keep in mind that gene scores are just an estimation of gene expression. Due to the way gene scores are calculated, they might not be entirely reliable for genes in gene-dense areas.

Box 23 **Calculating signature z-scores.**

**Table:** 

R code for calculating signature z-scores and overlaying them on the UMAP embedding	
*# Calculate signature z-scores (input: GRanges object)* archr_add_peak_signatures = **function**(proj, signature_list, signature_name){ *#signature_list: list of GRanges* *#signature_name: name string for the set of signatures* add_df_to_cellcoldata = **function**(pro, pheno_df, force=FALSE){ stopifnot(identical(rownames(pro@cellColData), rownames(pheno_df))) cnames = colnames(pheno_df) **for**(i **in** 1:ncol(pheno_df)){ pro = addCellColData(ArchRProj = pro, data=pheno_df[, i], name = cnames[i], cells = rownames(pro@cellColData), force = force) } **return**(pro) } **if**(length(signature_list)<2){ **stop**('Currently, only works if at least two signatures are provided') } **for**(i **in** seq_along(signature_list)){ names(signature_list[[i]]) = NULL } proj = addPeakAnnotations(ArchRProj = proj, regions = signature_list, name = signature_name, force = TRUE) method_use = "chromVAR" *#does only work with fixed width peaks* **if**(any(sapply(signature_list, **function**(x) length(unique(width(x)))) > 1)){ method_use = 'ArchR' } proj = addBgdPeaks(proj, force = T, method=method_use) proj = addDeviationsMatrix( ArchRProj = proj, peakAnnotation = signature_name, binarize = TRUE, bgdPeaks = getBgdPeaks(proj, method = method_use), force = TRUE ) dr_df = as.data.frame(proj@cellColData) sig_se = getMatrixFromProject(proj, paste0(signature_name, 'Matrix')) z_score_mat = t(assays(sig_se)[['z']]) z_score_mat = z_score_mat[match(rownames(dr_df),rownames(z_score_mat)), ] colnames(z_score_mat) = paste0('z_', colnames(z_score_mat)) stopifnot(identical(rownames(z_score_mat), rownames(dr_df))) dev_score_mat = t(assays(sig_se)[['deviations']]) dev_score_mat = dev_score_mat[match(rownames(dr_df),rownames(dev_score_mat)), ] colnames(dev_score_mat) = paste0('dev_', colnames(dev_score_mat)) stopifnot(identical(rownames(dev_score_mat), rownames(dr_df))) proj = add_df_to_cellcoldata(proj, z_score_mat, force=T) proj = add_df_to_cellcoldata(proj, dev_score_mat, force=T) **return**(proj) } signature_list = list( late_progenitor_tisTreg_sig = late_progenitor_tisTreg_GR, tisTreg_skin_sig = tisTreg_skin_GR ) proj_final = archr_add_peak_signatures(proj_final, signature_list, "signatures") *# Overlay signature z-scores on UMAP* p_tisTreg_skin_sig = plotEmbedding( ArchRProj = proj_final, colorBy = "cellColData", name = "z_tisTreg_skin_sig", embedding = "UMAP", plotAs = "points", size = 0.5 )

### Identifying marker features

Based on the gene scores, genes that can be leveraged to discriminate the cell state or type of any subset identified e.g. in a reduced dimensionality embedding, can be identified for either clusters (corresponding to cell types) or additional discrete covariates (e.g. tissue of origin, genotype, etc.). To this end, the group of cells is compared to a “background” group using a Wilcoxon rank-sum test (34) with multiple hypothesis test correction after Benjamini-Hochberg (35). For the background group, nearest neighbors in Euclidean space are selected after removing the bias introduced by the number of fragments per cell and the TSS Enrichment by applying the same relative scale to the variance of these two dimensions. Thus, the group of cells to identify marker genes for is compared to the cells that do not belong to this group themselves, but are the most similar cells in the dataset in terms of gene scores. This makes the calculated marker genes very specific for the group in this dataset.

Apart from the gene score matrix, other matrices like the tile matrix and the peak matrix (which will be introduced in the next paragraph) can be used as input to identify regions of accessible chromatin or peaks specific for a group of cells, respectively. See sections “2.1 Manual cluster-based annotation using prior-knowledge marker genes” (marker genes), “4.2 Identifying marker peaks” (marker peaks), and **Box 24**.

In addition to identifying marker features for a specific group, differential analysis can be performed on abovementioned matrices in order to identify differences between two groups, see section “4.3 Pairwise testing between groups” and **Box 25**.

Box 24 **Identifying marker features.**

**Table:** 

R code for identifying marker features	
*# Get marker features* markersGS = getMarkerFeatures( ArchRProj = proj, useMatrix = "GeneScoreMatrix", groupBy = "Clusters", bias = c("TSSEnrichment", "log10(nFrags)"), testMethod = "wilcoxon" )

Box 25 **Differential analysis.**

**Table:** 

R code for differential analysis	
*# Get differential peaks between tisTreg and Treg cell clusters* markerTest = getMarkerFeatures( ArchRProj = proj, useMatrix = "PeakMatrix", groupBy = "Clusters", testMethod = "wilcoxon", bias = c("TSSEnrichment", "log10(nFrags)"), useGroups = tisTreg_cluster, bgdGroups = tTreg_cluster )

### Creating pseudobulk replicates

Due to the sparse nature of scATAC-seq data, pseudobulk replicates have to be calculated in order to perform certain analyses, like peak calling and peak- and motif enrichment analysis. The creation of pseudobulk replicates is done as implemented in the original ArchR framework:

Cells are grouped by cluster, and pseudobulk replicates are created in a sample-aware fashion, if the cluster size and composition allows for it. It is important to note that pseudobulk replicates may be created in a sample-agnostic fashion and that cells may be sampled with replacement if the number of cells from each sample or the total cell number in a given cluster is lower than minCells x minReplicates, respectively (**Box 26**). For troubleshooting and recommendations see **Box 27**.

Box 26 **Computing pseudobulk replicates.**

**Table:** 

R code for computing pseudobulk replicates	
*# The key parameter here is groupBy, which defines the groups for which pseudo-bulk replicates should be made* proj = addGroupCoverages( ArchRProj = proj, groupBy = "Clusters", minCells = 40, maxCells = 500, minReplicates = 2, maxReplicates = 5, sampleRatio = 0.8 )

Box 27 **Troubleshooting and Recommendations.**

**Table:** 

Troubleshooting and Recommendations	
Description	Solution
Parameters for the generation of pseudobulk replicates	Parameters to tweak here are minCells, maxCells, minReplicates and maxReplicates, setting the min and max number of cells used for calculating pseudobulk replicates, and the min and max number of replicates calculated per cluster, respectively.
Sample-aware pseudobulk replicates	If you are interested in differences between samples as well as clusters, choose minCells in a way that allows pseudobulk replicates to be calculated in a sample-aware fashion.

### Peak calling using MACS2

On the pseudobulk data created above, we can now perform peak calling using MACS2 (36). This algorithm handles peak overlap between pseudobulk samples by iterative peak merging: Peaks are ordered by significance, and peaks overlapping with the peak of the highest significance are removed. This process is repeated until no peaks overlap. Peak calling is showcased in “4 Peak-Calling” and **Box 28**.

**Figure 10:**
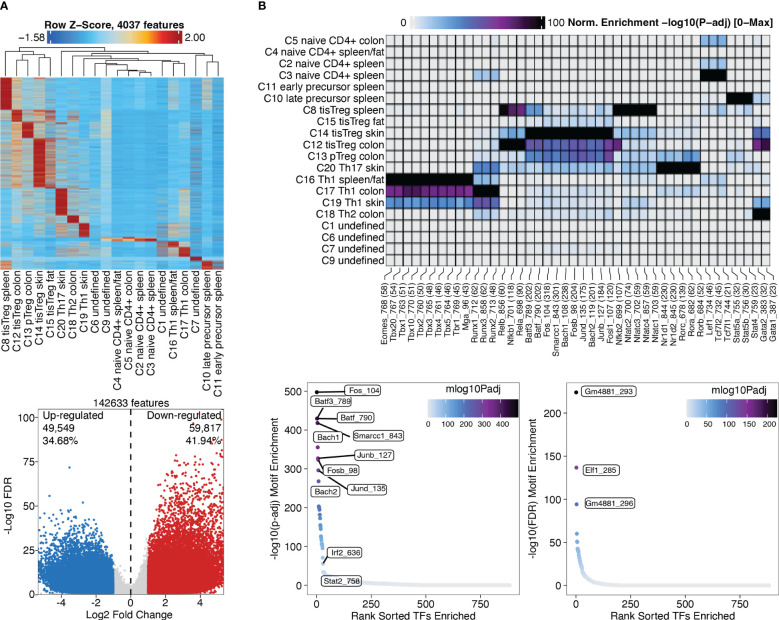
Marker peaks and differential peaks with TF motif enrichment. **(A)** Marker peaks grouped by clusters, with dendrogram indicating the overall similarity of clusters (top). Volcano plot showing differential peaks between tissue Treg cells and Treg cells from the spleen (bottom) **(B)** Motif enrichment in marker peaks grouped by clusters (top). Motifs enriched in tissue Treg cells compared to Treg cells (bottom left) and in Treg cells compared to tissue Treg cells (bottom right).

Marker peaks can then be identified in analogy to marker gene identification as described above, and as shown in section “4.2 Identifying Marker Peaks” of the script. A heatmap of the marker peaks for each cluster is shown in **Figure 10A**, top, with a dendrogram indicating the similarity of clusters in terms of marker peaks, as determined by Wilcoxon rank-sum test. Here we can nicely see how the cell types across tissue types cluster together. Differential peaks (see “4.3 Pairwise testing between groups”) between tissue Treg cells and Treg cells from the spleen are shown in **Figure 10A**, bottom.

Box 28 **Peak calling.**

**Table:** 

R code for calling peaks	
*# You can use the following function to search the path to Macs2* *# However, sometimes this might not work and you have to manually add the path* *# like it is shown in the second line.* pathToMacs2 = findMacs2() *# If you manually add the path, you have to change this line!* pathToMacs2 = "Path/to/Macs2" proj = addReproduciblePeakSet( ArchRProj = proj, groupBy = "Clusters", pathToMacs2 = pathToMacs2 ) *# add peak matrix to ArchRProject* proj = addPeakMatrix(proj)

### Motif and deviations enrichment, integrated with motif footprinting to identify upstream regulators of chromatin accessibility

After identifying marker peaks for the individual clusters, as well as differential peaks between two clusters of interest, we can now look for transcription factor (TF) motifs that are enriched in these peaks. This gives us insights into which transcription factors are active in a certain cell type, and into how different cell types differentially depend on certain transcription factors. To this end, a TF motif-by-peak matrix is created using motif annotations. The enrichment of certain motifs in marker peaks can then be analyzed (**Figure 10B**, top, **Box 29**, and “5.2 Motif enrichment in marker peaks”). Further, TF motifs in differential peaks can be analyzed (**Figure 10B**, bottom, **Box 30**, and “5.3 Motif enrichment in differential peaks”). Batf and associated AP-1 subunits are detected as enriched TF motifs in tissue Treg cells from different tissues, which nicely recapitulates the finding of Delacher et al. (37, 38). These are further the TF motifs, which are enriched in peaks differentially present in tissue Treg cells vs Treg cells from the spleen (**Figure 10B**).

Box 29 **Computing motif enrichment in marker peaks.**

**Table:** 

R code for computing motif enrichment in marker peaks	
*# We must first add these motif annotations to our ArchRProject; this* *# effectively creates a binary matrix where the presence of a motif in each peak* *# is indicated numerically* proj = addMotifAnnotations(ArchRProj = proj, motifSet = "cisbp", name = "Motif") *# We perform motif enrichment on our marker peaks* enrichMotifs = peakAnnoEnrichment( seMarker = markersPeaks, ArchRProj = proj, peakAnnotation = "Motif", cutOff = "FDR <= 0.1 & Log2FC >= 1" ) *# Plot these motif enrichments across all cell groups* heatmapEM = plotEnrichHeatmap(enrichMotifs, n = 10, transpose = TRUE ) *# Visualize* heatmapEM2 = ComplexHeatmap::draw(heatmapEM, heatmap_legend_side = "bot", annotation_legend_side = "bot", row_order = row_order )

Box 30 **Computing motif enrichment in differential peaks.**

**Table:** 

R code for computing motif enrichment in differential peaks between two clusters	
*# Create SummarizedExperiment object* motifsUp = peakAnnoEnrichment( seMarker = markerTest, ArchRProj = proj, peakAnnotation = "Motif", cutOff = "FDR <= 0.1 & Log2FC >= 0.5" ) *# Prepare data for plotting with ggplot* *# Create a simplified data.frame object containing the motif names, the corrected* *# p-values, and the significance rank* df_up = data.frame(TF = rownames(motifsUp), mlog10Padj = assay(motifsUp)[,1]) df_up = df_up[order(df_up$mlog10Padj, decreasing = TRUE),] df_up$rank = seq_len(nrow(df_up)) *# Plot rank-sorted TF motifs and color them by significance of their enrichment* ggUp = ggplot(df_up, aes(rank, mlog10Padj, color = mlog10Padj)) + geom_point(size = 1) + ggrepel::geom_label_repel( data = df_up[rev(seq_len(30)), ], aes(x = rank, y = mlog10Padj, label = TF), size = 1.5, nudge_x = 2, color = "black" ) + theme_ArchR() + ylab("-log10(P-adj) Motif Enrichment") + xlab("Rank Sorted TFs Enriched") + scale_color_gradientn(colors = paletteContinuous(set = "comet") )

**Figure 11:**
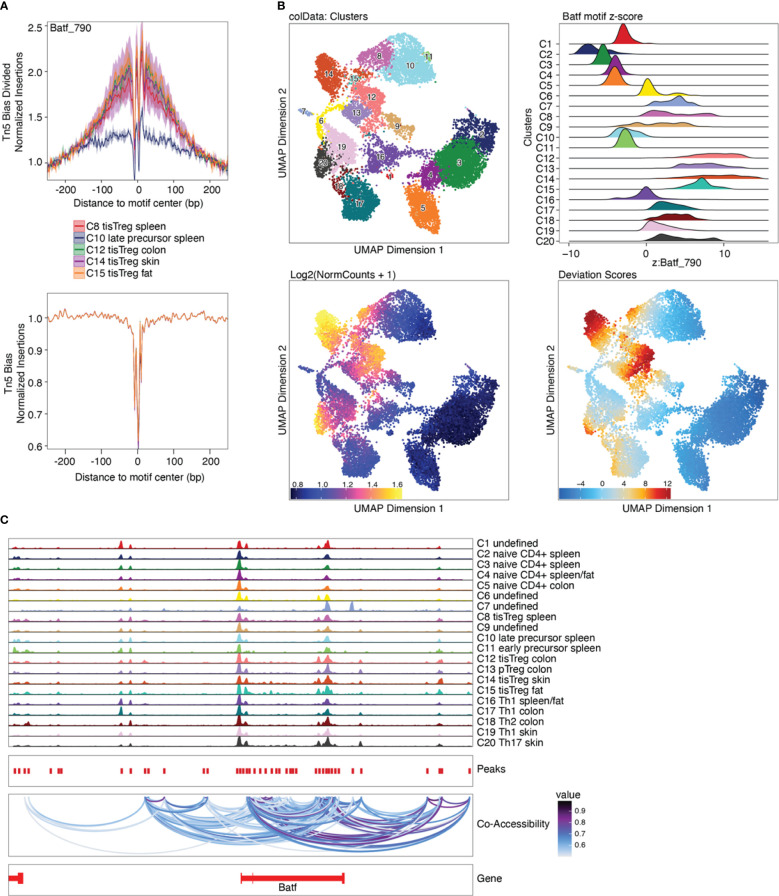
Motif footprinting, chromVAR, and co-accessibility. **(A)** Batf footprint in tissue Treg precursors (C10), tissue Treg cells from spleen (C8) and tissue Treg cells from non-lymphoid tissues (C12, C14, C15). **(B)** TF deviations computed using ChromVAR as ridge plot (top right) and Batf motif z-score as an overlay on the UMAP embedding (bottom right), next to the Batf gene score. **(C)** Co-accessibility analysis of Batf.

ChromVAR is an R package designed to infer TF-associated chromatin accessibility from scATAC-seq data on a single-cell basis, while accounting for the insertion bias introduced by the Tn5 transposase (33). For each cell, we calculated the deviation of accessibility of each motif compared to the expected motif accessibility based on all cells using ChromVAR, as well as the z-score, i.e. number of standard deviations a value deviates from the mean of the dataset. The deviations enrichment analysis implemented by ArchR is based on the ChromVAR approach, with adaptations for the processing of large datasets. The Batf motif z-score calculated with ChromVAR (**Box 31**) is shown in **Figure 11B**, with in increasing z-score from naïve CD4+ T cells, via early and late precursors to tissue Treg cells.

Box 31 **Computing ChromVAR deviations enrichment.**

**Table:** 

R code for predicting enrichment of TF activity on a per-cell basis using ChromVAR	
*# Add a set of background peaks; sample peaks based on similarity in GC-content and nFrags across all samples using the Mahalanobis distance* proj = addBgdPeaks(proj) *# Compute per-cell deviations across all of our motif annotations* proj = addDeviationsMatrix( ArchRProj = proj, peakAnnotation = "Motif", force = TRUE )

The prevalence of TF motifs of interest in a certain cell group can further be evaluated using ArchR’s *getFootprints* function (**Box 32**). Reads in all known binding locations of the respective TF are combined and insertion counts are plotted over the distance from the motif center. As can be seen in **Figure 11A**, insertion counts increase towards the motif center. At the motif center itself insertion counts drop, as DNA bases at the motif center are protected from transposition by TF binding. Footprinting is performed on the pseudobulk data generated above to achieve sufficient coverage. Footprint plots shown in **Figure 11A** indicate the prevalence of a certain TF footprint in a certain cluster or cell type. As expected, the footprint for Batf increases from tissue Treg precursor in the spleen (C10) to tissue Treg cells in the spleen (C8) to tissue Treg cells in non-lymphoid tissues (C12, C14, C15).

Box 32 **Calculating motif footprints.**

**Table:** 

R code for calculating motif footprints	
*# Obtain the positions of the relevant motifs* motifPositions = getPositions(proj, name = "Motif") *# This creates a GRangesList object where each TF motif is represented by a separate GRanges object* *# We can subset this GRangesList to a few TF motifs that we are interested in* motifs_fp = c("Foxp3", "Batf") markerMotifs_fp = unlist(lapply(motifs_fp, **function**(x) grep(x, names(motifPositions), value = TRUE) ) ) *# To accurately profile TF footprints, a large number of reads is required.* *# Therefore we will use the pseudobulk data stored as group coverages calculated above.* *# Compute footprints for the subset of marker motifs defined above:* seFoot = getFootprints( ArchRProj = proj, positions = motifPositions[markerMotifs_fp], groupBy = "Clusters" )

### Analyzing co-accessibility of genomic regions

To find which peaks are often accessible together, co-accessibility analysis can be performed on peaks of single cells across clusters. A typical use case for this approach would be to identify the regions enriched in regulatory elements (such as promoters and enhancers) which are likely to operate together. Since peaks can be very similar within a cell type, co-accessibility analysis will also find a correlation for peaks specific for a cell type. Thus, co-accessibility analysis does not allow for identification of regulatory relationships (see **Box 33**). **Figure 11C**, section “8 Co-accessibility analysis” of the script, **Box 34**.

Box 33 **Troubleshooting and Recommendations.**

**Table:** 

Troubleshooting and Recommendations	
Description	Solution
Correlation/ regulatory relationship	Besides peaks being co-accessible as result of a regulatory relationship, peaks are also often co-accessible in one cell type compared to other cell types. The latter case simply is correlation, not causation, therefore co-accessibility analysis does not allow for the identification of regulatory relationships.

Box 34 **Co-accessibility analysis.**

**Table:** 

R code for co-accessibility analysis	
*# Calculate co-accessibility* proj_final = addCoAccessibility( ArchRProj = proj_final, reducedDims = "IterativeLSI" ) *# Retrieve co-accessibility information via the getCoAccessibility() function* cA = getCoAccessibility( ArchRProj = proj_final, corCutOff = 0.5, resolution = 1000, returnLoops = TRUE ) *# Plot browser tracks of co-accessibility for our marker genes* markerGenes = "Batf" p_cA = plotBrowserTrack( ArchRProj = proj_final, groupBy = "Clusters", geneSymbol = markerGenes, upstream = 50000, downstream = 50000, loops = getCoAccessibility(proj_final) )

### Analyzing gene and motif scores along pseudotime

**Figure 12:**
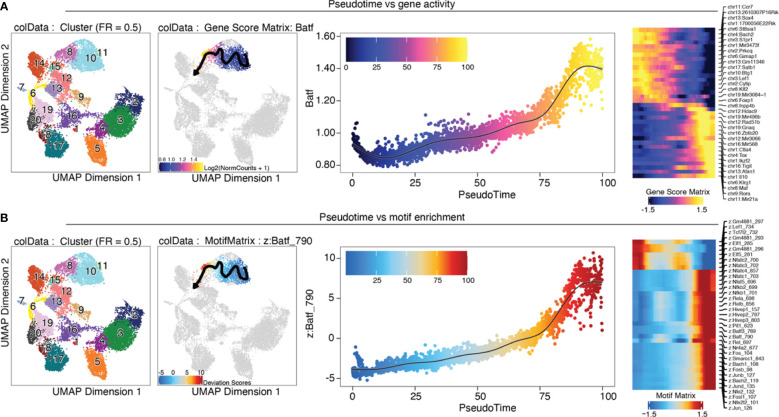
Trajectory analysis. **(A)** from left to right: UMAP colored by clusters; trajectory from C11 early progenitors via C10 late progenitors and C8 tissue Treg cell in the spleen to C15 VAT tissue Treg cells; Batf gene score is shown over pseudotime; heatmap of top variable features over pseudotime **(B)** Analogous to **(A)**, motif enrichment is shown over pseudotime.

Trajectory analysis is very useful for analyzing gene expression or motif enrichment along pseudotime, as a proxy of the “real” time over continuous processes such as development and differentiation. The trajectory analysis approach implemented in ArchR needs prior knowledge on developmental stages of the cells. In our case we know that tissue Treg cells develop from early progenitors via late progenitors and tissue Treg cell in the spleen to tissue Treg cells in non-lymphoid tissues (39). Along the user-defined backbone (in our case, the set of clusters C11, C10, C8, C15), a pseudotime vector is calculated as follows: 1) the mean coordinates for each cluster are calculated in the LSI subspace, and the top 5% of cells closest to the mean coordinates will be kept. 2) A pseudotime vector is calculated from the distance of each cell from a cluster to the mean coordinates of the cluster that comes next in the user-defined backbone, and a trajectory is fitted. 3) For all cells in the user-defined clusters, the nearest point to the trajectory in Euclidian space is found, and cells are aligned to the trajectory. Gene scores or motif enrichment can then be plotted along the trajectory (**Box 35**, section “9 Trajectory Analysis” of the script). In **Figure 12** we plotted the Batf gene score (A) and motif enrichment (B) along pseudotime from early and late precursors to tissue Treg cells in the spleen, to VAT tissue Treg cells. We observe a steady increase of both the gene score and the motif enrichment over pseudotime, which is what we would expect, considering that Batf orchestrates the tissue repair program. Further, heatmaps of gene and TF activity along pseudotime of the top variable genes or motifs are shown. Trajectory analysis without prior knowledge can be performed using Slingshot (40) or Monocle 3 (41–43).

Box 35 **Trajectory analysis.**

**Table:** 

R code for trajectory analysis	
*# Create user-defined trajectory backbone* Treg_trajectory_VAT = c("C11", "C10", "C8", "C15") *# Create the trajectory* proj_final = addTrajectory( ArchRProj = proj_final, name = "Treg_trajectory_VAT", groupBy = "Clusters", trajectory = Treg_trajectory_VAT, embedding = "UMAP", force = TRUE ) *# Exclude cells with NA values because these are not part of the trajectory* proj_final$Treg_trajectory_VAT[!is.na(proj_final$Treg_trajectory_VAT)] *# Overlay pseudotime values on UMAP embedding* Treg_trajectory_VAT_p = plotTrajectory(proj_final, trajectory = "Treg_trajectory_VAT", colorBy = "cellColData", name = "Treg_trajectory_VAT", plotAs = "points" ) *# Plot gene scores and motif enrichment along Treg trajectory* Treg_traj_VAT_p_Batf = plotTrajectory(proj_final, trajectory = "Treg_trajectory_VAT", colorBy = "GeneScoreMatrix", name = "Batf", continuousSet = "horizonExtra", plotAs = "points" ) Treg_traj_VAT_p_Batf = plotTrajectory(proj_final, trajectory = "Treg_trajectory_VAT", colorBy = "MotifMatrix", name = "Batf_790", continuousSet = "horizonExtra", plotAs = "points" ) *# Visualize changes in features (MotifMatrix, GeneScoreMatrix) across* *# pseudo-time using heatmaps.* *# varCutOff (variance quantile cut-off) can be adjusted to set the top variable* *# features across the trajectory* *# Treg pseudotime GeneScoreMatrix:* Treg_trajGSM = getTrajectory(ArchRProj = proj, name = "Treg_trajectory_VAT", useMatrix = "GeneScoreMatrix", log2Norm = FALSE ) *# Plot:* Treg_trajGSM_p = plotTrajectoryHeatmap(Treg_trajGSM, pal = paletteContinuous(set = "horizonExtra"), varCutOff = 0.9 )

## Conclusion

In this article, we provide an end-to-end solution covering every step from the isolation of high-quality CD4+ T cells from murine tissues, via scATAC-seq library generation and sequencing, to data pre-processing and advanced bioinformatic analysis. We draw attention to possible pitfalls and give recommendations regarding delicate steps. While our method is focused on the chromatin accessibility for tissue Treg cells, we can anticipate the omics landscape will expand in the coming years, obtaining simultaneously multi-omics and spatial profiles for the system under investigation. Moreover, our bioinformatics workflow can smoothly be reproduced, expanded, and adapted to other scenarios, empowering researchers to perform comprehensive and complex workflows.

## Data availability statement

Publicly available datasets analyzed in this article can be found at the Gene Expression Omnibus database, with accession code GSE156112. The code generated throughout this article is available at the GitHub repository https://github.com/imbeimainz/scATACseq_TissueTcells. The rendered HTML notebooks accompanying the analyses presented can be found on Zenodo (https://zenodo.org/record/8160122).

## Author contributions

Conceptualization and design: MD and FM. Manuscript writing: KLB and SSH. Analysis pipeline: KLB, ASN, MS and NB. Manuscript revision: all authors. All authors contributed to the article and approved the submitted version.
